# The Association Between Periconceptual Maternal Dietary Patterns and Miscarriage Risk in Women With Recurrent Miscarriages: A Multicentre Cohort Study

**DOI:** 10.1111/1471-0528.18022

**Published:** 2024-11-26

**Authors:** Yealin Chung, Pedro Melo, Christina Easter, Malcolm J. Price, Rima Dhillon‐Smith, Siobhan Quenby, Adam Devall, Arri Coomarasamy

**Affiliations:** ^1^ Tommy's National Centre for Miscarriage Research, Institute of Metabolism and Systems Research, College of Medical and Dental Sciences University of Birmingham Edgbaston UK; ^2^ CARE Fertility Birmingham Edgbaston UK; ^3^ Nuffield Department of Women's & Reproductive Health, Women's Centre, John Radcliffe Hospital University of Oxford Oxford UK; ^4^ Institute of Applied Health Research University of Birmingham Edgbaston UK; ^5^ Department of Public Health Canadian University Dubai Dubai UAE; ^6^ Division of Biomedical Sciences, Warwick Medical School University of Warwick Warwick UK; ^7^ Tommy's National Centre for Miscarriage Research University Hospitals Coventry and Warwickshire NHS Trust Coventry UK

**Keywords:** maternal diet, recurrent miscarriage, recurrent pregnancy loss

## Abstract

**Objective:**

To examine the association between periconceptual maternal diet and miscarriage risk among women with recurrent miscarriages.

**Design:**

Prospective multicentre cohort study (Tommy's Net).

**Setting:**

Three university hospital research centres in the United Kingdom.

**Population:**

1035 women with a baseline history of two or more miscarriages.

**Methods:**

We analysed baseline dietary data from a 10‐item Food Frequency Questionnaire (FFQ). For individual food category analyses, we used multivariable Poisson regression following adjustment for maternal confounders and paternal dietary patterns. For whole diet analyses, ordinal principal component analysis (PCA) was used to identify common dietary patterns. Results were presented as relative risks (RR) with 95% confidence intervals (CI) and accompanying *p*‐values.

**Main Outcome Measures:**

Miscarriage rate, defined as the rate of spontaneous pregnancy loss (< 24 weeks of gestation) relative to the total number of pregnancies (miscarriages and live births).

**Results:**

High consumption of fruit and nuts (almonds and walnuts) was associated with lower miscarriage risk (fruit 226/662 (34.1%) vs. 38/77 (49.4%), RR 0.66, 95% CI 0.51 to 0.85, *p* = 0.001; nuts 47/152 (30.9%) vs. 220/613 (35.9%), RR 0.73, 95% CI 0.54 to 0.98, *p* = 0.039). High red meat intake was associated with a possible increase in miscarriage risk (6/12 (50.0%) vs. 165/469 (35.2%), RR 1.86, 95% CI 1.10 to 3.16, *p* = 0.022). The association with miscarriage risk was unclear for other food groups, including fresh vegetables, white meat, fish, dairy, eggs, soya and chocolate, due to imprecise point estimates. Through PCA, we identified three data‐derived dietary patterns. Yet, no distinct relationship emerged between these dietary patterns and miscarriage risk.

**Conclusions:**

A maternal diet rich in fresh fruits and nuts is associated with a lower miscarriage risk among women with a history of recurrent miscarriage.

**Trail Registration:**

Tommy's Net (ISRCTN17732518) https://www.isrctn.com/ISRCTN17732518. Analysis plan (OSF zp7cs) https://osf.io/zp7cs.

## 1 Introduction

The periconceptual period represents a pivotal window during which crucial reproductive stages unfold, from folliculogenesis to embryo implantation and the establishment of early pregnancy. Numerous national and international guidelines highlight the need to optimise lifestyle factors during this sensitive period [1, 2, 3, 4, 5]. This stems from evidence suggesting that lifestyle factors may alter gametogenesis and embryo quality, in turn contributing to an increased risk of pregnancy complications [6]. Miscarriage, defined as the spontaneous loss of a pregnancy before viability, constitutes the most common of these complications, occurring in 1 in 6 clinically recognised pregnancies [7].

Maternal diet influences pregnancy outcomes, the health of the offspring and future maternal health outcomes [8, 9, 10, 11, 12, 13]. This knowledge is supported by evidence that maternal nutritional deficiencies increase the risk of pregnancy complications including congenital heart defects, miscarriage and neural tube defects [14, 15, 16]. Maternal nutrition has also been shown to modulate the epigenetic landscape of the offspring, affecting its susceptibility to metabolic and disease risk in later life [17, 18]. In addition, poor nutritional status before or during pregnancy has been associated with a heightened risk of maternal development of non‐communicable diseases in the post‐reproductive years [19]. However, there is uncertainty about what constitutes a healthy diet and nutritional status in pregnancy. Recent data suggest that a nuanced approach to dietary balance and nutritional composition may offer substantial advantages over an exclusive focus on correcting nutrient deficiencies [20, 21, 22].

A recent systematic review and meta‐analyses [23] found evidence that a periconceptual maternal diet abundant in fruit, vegetables, seafood, dairy, eggs and grain may be associated with decreased miscarriage odds. Evaluating diet holistically, patterns containing foods perceived to be healthy by the trialists or those high in antioxidant components appeared to be associated with reduced miscarriage odds. Notably, in this review, most of the evidence was derived from studies focusing on generally healthy reproductive‐aged women rather than on those experiencing recurrent pregnancy loss.

The present study aimed to address this existing evidence gap by evaluating the association between maternal dietary choices and the risk of further miscarriages in women with a history of recurrent pregnancy loss.

## 2 Methods

The reporting of our findings adhered to the Strengthening the Reporting of Observational Studies in Epidemiology (STROBE) guidelines [24].

### 2.1 Study Design

We analysed data from Tommy's Net (ISRCTN17732518), a dedicated electronic database for miscarriage‐related research. This database is an integral component of a prospective cohort study designed to investigate pregnancy outcomes in individuals and couples who have experienced miscarriages. It serves as a secure, centralised storage system that consolidates research data from three university hospital research centres in the United Kingdom: Birmingham, Coventry and London. Together, these centres comprise the Tommy's National Centre for Miscarriage Research. The primary objectives of this e‐repository are multifaceted, including but not limited to facilitating population‐based epidemiological studies on miscarriage and aiding in the development and validation of tests and predictive models for determining pregnancy outcomes. Recruitment for the study commenced in 2017 and remains ongoing.

### 2.2 Participants

Eligible participants are couples who have experienced one or more pregnancy losses and are currently seeking specialist services at the participating sites. Upon recruitment, we collect baseline information, including sociodemographic data, medical and reproductive history, as well as diet and lifestyle information. Participants then consult with a clinician who reviews their history, advises on further investigations and management and assesses eligibility for other studies and trials. Follow‐up appointments are offered to review investigation results and plan for future pregnancies.

Every 6 months, participants update information on their subsequent pregnancy outcomes. They achieve this by directly inputting data into an anonymised online system, accessible through a link sent via mobile phone text. We use the term ‘index pregnancy’ to refer to the first pregnancy occurring during the follow‐up period. Initially hosted by the University Hospitals Coventry and Warwickshire NHS Trust, the online system has transitioned to the University of Birmingham as of 2023. In addition, an authorised researcher systematically gathers and inputs outcome data into the system, which involves collecting information during hospital appointments, from patient notes, or through telephone calls.

### 2.3 Exposure Assessment

#### 2.3.1 Diet Assessment

Dietary intake was estimated using a Food Frequency Questionnaire (FFQ) developed specifically for the Tommy's Net study (Data S2). The FFQ was designed to estimate the routine dietary habits of participants at the point of recruitment. The questionnaire covers 10 distinct food categories: fresh fruit, fresh vegetables, red meat, white meat, fish, dairy products, eggs, soya products, chocolate and nuts (almonds or walnuts). These categories were judiciously selected to balance routine consumption and their known association with health outcomes, ensuring the questionnaire's relevance to typical diets and broader nutritional research. The participants were instructed to report the frequency, on a scale of 0–7 days/week, at which they consumed each food group. By focusing on a limited yet diverse range of food groups, the FFQ balances brevity and comprehensiveness, thereby minimising participant burden [25, 26].

#### 2.3.2 Covariate Assessment

Tommy's Net collects comprehensive baseline characteristics and follow‐up parameters for all participants from the self‐reported baseline questionnaire. In selecting covariates for the statistical model, we used a directed acyclic graph (DAG) [27], developed using the web‐based software DAGitty [28]. The covariate selection process was guided by existing evidence of factors associated with dietary choice and miscarriage risk. Figure S1 presents the simplified version of the DAG. The full version of the DAG can be accessed at https://dagitty.net/mTFWRU6e2. The minimal sufficient adjustment set we used for the statistical analyses included maternal age, ascertained from the mother's age at the time of conception of the index pregnancy, derived from her self‐reported date of birth; maternal body mass index (BMI), derived from weight and height measurements taken at recruitment (kg/m^2^); maternal ethnicity, categorised as White, Mixed, Asian, Black or other; alcohol consumption and smoking status, captured as binary responses (either ‘yes’ or ‘no’ to current alcohol consumption or cigarette smoking); and the number of previous live births and miscarriages, expressed as numerical variables. Additionally, we factored in linked paternal dietary patterns during individual food category analyses by adjusting for the corresponding paternal consumption of the specific food category being assessed. The assessment of paternal diet mirrored the method described for maternal dietary assessment, and adjustment for this is further described in the sensitivity analysis.

### 2.4 Outcome Ascertainment

The primary outcome was the miscarriage rate, defined as the spontaneous loss of a pregnancy up to 24 weeks of gestation relative to the total number of pregnancies (i.e., both miscarriages and live births). This definition excludes ectopic or molar pregnancies and terminations for any reason. Within Tommy's Net, we gathered data regarding whether the loss was biochemical and confirmed by ultrasound, among other factors. This included the estimated gestational age at diagnosis and the method used to manage the miscarriage. We adopted a broader definition encompassing all spontaneous pregnancy losses regardless of ultrasound visualisation. This approach aligns with the recommendations from an international consensus development study on core outcome sets for studies in miscarriage management and prevention [29]. To confirm self‐reported miscarriages, we consulted medical records where available to ensure the accuracy and validity of patient‐reported outcomes. If medical records were unavailable or if there was no correspondence between records and self‐reported outcomes, participants were sent reminder texts prompting them to provide updates regarding any subsequent pregnancies and their outcomes. Further outcome data were obtained during viability ultrasound scan visits.

### 2.5 Study Size

As of 5 March 2023, the database contained information on 3254 women and their 2530 linked partners. For this analysis, we excluded 369 women who, at enrolment, did not have a history of recurrent miscarriage, defined as 2 or more pregnancy losses at < 24 weeks of gestation. Additionally, 1360 individuals who did not report subsequent follow‐up pregnancies were excluded. Among participants who reported subsequent pregnancy outcomes, we further excluded 156 participants whose index pregnancy did not result in relevant outcomes of interest, such as ectopic or molar pregnancies, termination for any reasons or a loss beyond 24 weeks of gestation. This exclusion also encompasses patients with ongoing pregnancies, whose outcomes were yet to be determined. Moreover, 334 patients who did not provide maternal dietary information were excluded. The final analysis sample comprised 1035 women, with paternal dietary information available for 889 of these.

### 2.6 Statistical Methods

Baseline characteristics were analysed as follows: categorical variables were reported using frequencies and percentages, while continuous variables were displayed using means and standard deviations. The characteristics of the cohort were stratified by primary exposures of interest, which were grouped into tertiles for the 10 food categories: low intake (0–1 days/week), moderate intake (2–4 days/week) and high intake (5–7 days/week). Although we considered treating food exposure as a continuous variable, the skewed distribution and small sample sizes within certain intake groups made this approach challenging. Categorising the data into tertiles minimised the risk of overfitting while capturing meaningful differences in exposure levels, thus supporting more reliable analyses.

#### 2.6.1 Primary Analysis

The primary analysis of the relationship between maternal diet and miscarriage risk was conducted in two distinct stages. Firstly, an individual food category analysis, focusing on the individual association between specific food category consumption and miscarriage risk, was conducted. For this analysis, food intake was categorised into tertiles as described previously. Multivariable Poisson regression with robust standard errors was used to account for potential confounders described above. Results were presented using relative risks (RR), 95% confidence intervals (CI) and *p*‐values. The second stage involved an overall dietary pattern analysis, investigating the association between whole dietary pattern and miscarriage risk using an a posteriori method. We used ordinal principal component analysis (PCA) based on the polychoric correlation to identify common dietary patterns, highlight the most important variables, reduce dimensionality and address issues of collinearity of the multivariable dietary dataset. The suitability of our dataset for PCA was confirmed with the calculation of the Kaiser–Meyer–Olkin (KMO) measure of sampling adequacy, applying an overall threshold of 0.6. We retained dietary patterns that satisfy the Kaiser rule (eigenvalue above 1) and lie above the elbow inflection point identified in the scree plot analysis. Following this, an adherence score was determined for each statistically identified dietary pattern. These scores were then categorised into tertiles, ranging from the lowest to the highest prediction score. Ultimately, the prediction scores for the dietary patterns were evaluated as potential predictors of miscarriage risk using a Poisson regression model with robust standard errors. Results were presented as RR with 95% CI and accompanying *p*‐values. To address the uncertainty inherent in the PCA and the subsequent regression models, we performed bootstrapping of the entire process as an additional analysis.

#### 2.6.2 Subgroup Analysis

In addition to our primary analyses, we conducted several pre‐defined subgroup analyses. The subgroup analyses are delineated as follows: (1) age, grouped into < 35 and ≥ 35 years, given the evidence of adverse outcomes for women aged 35 and above [7]; (2) BMI categories, including underweight (BMI < 19 kg/m^2^), normal weight (BMI 19–25 kg/m^2^) and overweight or obesity (BMI ≥ 25 kg/m^2^), based on outcomes correlating with BMI extremes [7]; (3) ethnicity, including White, Mixed, Asian, Black and other, because of evidence suggesting that certain ethnic backgrounds, such as Asian or Black, face worse pregnancy outcomes [7]; (4) previous pregnancy history, grouping mothers according to the number of previous miscarriages (2, 3, 4, and 5 or more); (5) alcohol consumption, analysed by grouping alcohol drinkers and non‐drinkers; and (6) smoking status, analysed by grouping smokers and non‐smokers.

#### 2.6.3 Sensitivity Analysis and Missing Data

To address missing data, we followed the methods outlined by Jakobsen and colleagues [30]. Our primary analyses were conducted on datasets with complete maternal dietary records and corresponding pregnancy outcomes. Upon assessing the extent of missing data, we identified that approximately 16% of the dataset had missing values for key maternal covariates and paternal dietary information. These data were primarily determined to be Missing at Random (MAR). Therefore, we conducted sensitivity analyses to evaluate how different statistical methods for handling missing data might influence our conclusions. Our sensitivity analysis involved a multi‐step approach. First, we conducted a complete‐case analysis using datasets that included only participants with complete dietary data for both maternal and paternal records. This approach allowed us to assess whether excluding cases with missing data could bias our primary results. Next, we implemented two types of multiple imputation analyses using the chained equations. The first imputation addressed missing values for key maternal covariates and paternal dietary information. The second imputation extended this by including participants previously excluded due to missing maternal dietary data and imputing these missing values. Multiple imputation replaces each missing value with a set of plausible values derived from the distribution of the observed data, generating several complete datasets that represent a range of possible scenarios. Each of these datasets is analysed separately, and the results are then combined to produce a single set of estimates that account for the uncertainty due to missing data. The pooling of results is typically performed using Rubin's rules, which adjust the standard errors to reflect the variability among the imputed datasets. By comparing estimates from both the complete‐case and multiple imputation analyses, we were able to evaluate the impact of missing data on our findings and reinforce the reliability of our conclusions irrespective of how the missing data are handled. Additional details regarding the extent of missing data can be found in Table S3.

Finally, to address potential unmeasured confounding in our primary findings, we calculated E‐values as part of the sensitivity analysis. E‐values quantify the minimum strength of association that an unmeasured confounder would need to have with both the exposure and outcome to explain away the observed association.

## 3 Results

Table 1 provides an overview of baseline participant characteristics for the stratified cohorts. On average, the interval between entry and conception was 175 days.

**TABLE 1:** Characteristics of Tommy's Net cohort with recurrent miscarriages by maternal food category exposure tertiles.

Baseline characteristics	Low intake (0–1 days per week)	Moderate intake (2–4 days per week)	High intake (5–7 days per week)
	Mean ± SD number/total (%)	Mean ± SD number/total (%)	Mean ± SD number/total (%)
Fresh fruit			
Maternal age at conception (years)	32.8 ± 5.7	33.1 ± 5.2	35.0 ± 4.5
BMI (kg/m^2^)	26.7 ± 5.4	26.6 ± 5.8	26.1 ± 5.4
White ethnicity	67/77 (87)	247/296 (83)	576/662 (87)
Non‐smoker at baseline	65/77 (84)	262/295 (89)	632/661 (96)
Non‐alcohol drinker at baseline	41/77 (53)	144/295 (49)	309/662 (47)
Number of previous live birth at baseline	0 ± 1	0 ± 1	0 ± 1
Number of previous miscarriages at baseline	4 ± 2	3 ± 2	3 ± 2
Fresh vegetables			
Maternal age at conception (years)	31.2 ± 5.6	32.9 ± 5.5	34.9 ± 4.5
BMI (kg/m^2^)	27.0 ± 5.8	26.7 ± 5.9	26.1 ± 5.4
White ethnicity	36/42 (86)	179/226 (79)	675/767 (88)
Non‐smoker at baseline	32/42 (76)	199/226 (88)	728/765 (95)
Non‐alcohol drinker at baseline	25/42 (60)	130/226 (58)	339/766 (44)
Number of previous live birth at baseline	1 ± 1	0 ± 1	0 ± 1
Number of previous miscarriages at baseline	3 ± 1	4 ± 2	3 ± 2
Red meat			
Maternal age at conception (years)	34.4 ± 5.0	34.2 ± 4.8	31.4 ± 4.0
BMI (kg/m^2^)	25.8 ± 5.2	26.6 ± 5.8	26.8 ± 5.8
White ethnicity	379/469 (81)	504/554 (91)	7/12 (58)
Non‐smoker at baseline	433/467 (93)	517/554 (93)	9/12 (75)
Non‐alcohol drinker at baseline	251/468 (54)	237/554 (43)	6/12 (50)
Number of previous live birth at baseline	0 ± 1	0 ± 1	1 ± 1
Number of previous miscarriages at baseline	3 ± 1	4 ± 2	4 ± 2
White meat			
Maternal age at conception (years)	34.8 ± 4.8	34.1 ± 4.9	34.1 ± 5.0
BMI (kg/m^2^)	26.1 ± 5.5	26.2 ± 5.4	27.0 ± 6.1
White ethnicity	220/272 (81)	578/659 (88)	92/104 (88)
Non‐smoker at baseline	257/271 (95)	609/658 (93)	93/104 (89)
Non‐alcohol drinker at baseline	138/271 (51)	307/659 (47)	49/10 (490)
Number of previous live birth at baseline	0 ± 1	0 ± 1	0 ± 1
Number of previous miscarriages at baseline	3 ± 1	4 ± 2	4 ± 2
Fish			
Maternal age at conception (years)	33.7 ± 4.9	35.5 ± 4.7	34.6 ± 4.2
BMI (kg/m^2^)	26.5 ± 5.7	25.7 ± 5.1	27.1 ± 6.5
White ethnicity	593/691 (86)	285/325 (88)	12/19 (63)
Non‐smoker at baseline	629/690 (91)	312/324 (96)	18/19 (95)
Non‐alcohol drinker at baseline	348/690 (50)	137/325 (42)	9/19 (47)
Number of previous live birth at baseline	0 ± 1	0 ± 1	0 ± 1
Number of previous miscarriages at baseline	3 ± 2	3 ± 2	3 ± 2
Dairy products			
Maternal age at conception (years)	34.1 ± 4.7	34.2 ± 5.6	34.4 ± 4.8
BMI (kg/m^2^)	27.0 ± 6.0	26.5 ± 5.7	26.0 ± 5.3
White ethnicity	178/210 (85)	135/168 (80)	577/657 (88)
Non‐smoker at baseline	193/210 (92)	154/168 (92)	612/655 (93)
Non‐alcohol drinker at baseline	124/210 (59)	89/168 (53)	281/656 (43)
Number of previous live birth at baseline	0 ± 1	0 ± 1	0 ± 1
Number of previous miscarriages at baseline	3 ± 2	4 ± 2	3 ± 2
Eggs			
Maternal age at conception (years)	33.6 ± 5.0	34.8 ± 4.7	35.4 ± 4.5
BMI (kg/m^2^)	26.3 ± 5.8	26.3 ± 5.3	26.2 ± 5.3
White ethnicity	428/497 (86)	376/433 (87)	86/105 (82)
Non‐smoker at baseline	458/496 (92)	401/432 (93)	100/105 (95)
Non‐alcohol drinker at baseline	264/497 (53)	188/432 (44)	42/105 (40)
Number of previous live birth at baseline	0 ± 1	0 ± 1	1 ± 1
Number of previous miscarriages at baseline	3 ± 2	3 ± 2	4 ± 2
Soya products			
Maternal age at conception (years)	34.2 ± 5.0	35.2 ± 4.6	34.4 ± 4.0
BMI (kg/m^2^)	26.4 ± 5.5	25.6 ± 5.3	26.3 ± 5.4
White ethnicity	774/896 (86)	84/102 (82)	32/37 (86)
Non‐smoker at baseline	830/895 (93)	94/101 (93)	35/37 (95)
Non‐alcohol drinker at baseline	436/896 (49)	44/101 (44)	14/37 (38)
Number of previous live birth at baseline	0 ± 1	0 ± 1	0 ± 0
Number of previous miscarriages at baseline	3 ± 2	3 ± 1	4 ± 2
Chocolate			
Maternal age at conception (years)	34.6 ± 4.8	34.1 ± 4.9	34.1 ± 5.0
BMI (kg/m^2^)	26.5 ± 5.6	26.2 ± 5.4	26.1 ± 5.5
White ethnicity	293/356 (82)	400/456 (88)	197/223 (88)
Non‐smoker at baseline	330/356 (93)	420/454 (93)	209/223 (94)
Non‐alcohol drinker at baseline	193/356 (54)	197/455 (43)	104/223 (47)
Number of previous live birth at baseline	0 ± 1	0 ± 1	1 ± 1
Number of previous miscarriages at baseline	3 ± 2	4 ± 2	3 ± 2
Nuts (almonds or walnuts)			
Maternal age at conception (years)	33.4 ± 5.0	35.4 ± 4.7	35.8 ± 4.2
BMI (kg/m^2^)	26.8 ± 5.7	25.9 ± 5.2	24.6 ± 4.7
White ethnicity	537/613 (88)	224/270 (83)	129/152 (85)
Non‐smoker at baseline	559/613 (91)	251/268 (94)	149/152 (98)
Non‐alcohol drinker at baseline	306/613 (50)	121/269 (45)	67/152 (44)
Number of previous live birth at baseline	0 ± 1	0 ± 1	0 ± 1
Number of previous miscarriages at baseline	3 ± 2	4 ± 2	3 ± 1

### 3.1 Individual Food Category Analysis

**FIGURE 1:**
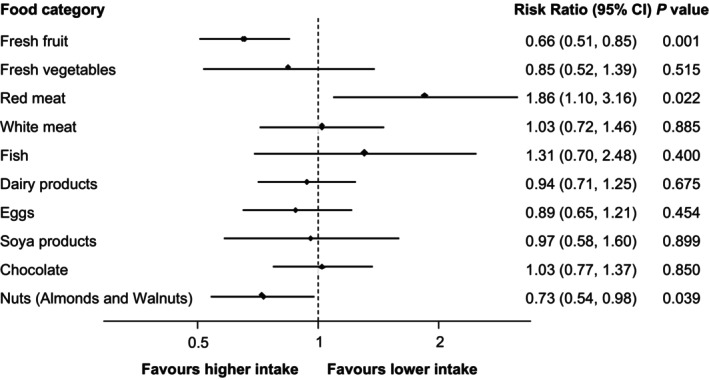
Individual maternal food categories associations with miscarriage risk (lower vs. higher intake). A plot of miscarriage risk ratios, 95% confidence intervals (CI), and *p* values using Poisson regression with full adjustment for maternal factors and paternal dietary patterns. The *y*‐axis lists each maternal food category. The *x*‐axis shows the miscarriage risk ratios for each category comparing lower intake (0–1 days per week) to higher intake (5–7 days per week). The black center point shows the risk ratio for each category and the error bars show the 95% CI about this value. A plot of miscarriage risk ratios, 95% confidence intervals (CI), and *p* values using Poisson regression with full adjustment for maternal factors and paternal dietary patterns. The *y*‐axis lists each maternal food category. The *x*‐axis shows the miscarriage risk ratios for each category comparing lower intake (0–1 days per week) to higher intake (5–7 days per week). The black center point shows the risk ratio for each category and the error bars show the 95% CI about this value.

Figure 1 presents point estimates, 95% CI and *p*‐values for Poisson regression analysis comparing the low and high‐intake groups. The multivariable analysis showed that compared to a low fresh fruit intake, high intake was associated with a lower miscarriage risk (226/662 (34.1%) vs. 38/77 (49.4%), RR 0.66, 95% CI 0.51 to 0.85, *p* = 0.001). The analysis also showed an 86% higher miscarriage risk with high red meat consumption (6/12 (50.0%) vs. 165/469 (35.2%), RR 1.86, 95% CI 1.10 to 3.16, *p* = 0.022) compared to low consumption. Nut consumption followed a contrasting trend showing a 27% lower miscarriage risk with high consumption (47/152 (30.9%) vs. 220/613 (35.9%), RR 0.73, 95% CI 0.54 to 0.98, *p* = 0.039) compared to low. For fresh vegetables and eggs, the point estimates indicated a possible reduction in miscarriage risk but the 95% CI was imprecise, making the association with miscarriage risk unclear. For white meat, dairy products and chocolate, the point estimates are close to one with confidence intervals leading to inconclusive results. Finally, the evidence showed that high fish consumption may be linked with an increased miscarriage risk, although wide confidence intervals resulted in imprecision and uncertainty. Table 2 presents proportions, point estimates, 95% CI, *p*‐values and E‐values for both univariable and multivariable analyses. The results show broad consistency across these analyses.

**TABLE 2:** Poisson regression analysis of individual maternal food categories in Tommy's Net recurrent miscarriage cohort.

Food items (days per week)	Miscarriage number	Total pregnancy number	Miscarriage rate (%)	Univariable^a^ RR (95% CI)	*p*	Multivariable^b^ RR (95% CI)	*p*	*E* ^c^
Fresh fruit								
Low (0–1)	38	77	49.4	Reference		Reference		
Mod (2–4)	95	296	32.1	0.65 (0.49–0.86)	0.003	0.61 (0.46–0.82)	0.001	2.663
High (5–7)	226	662	34.1	0.69 (0.54–0.89)	0.004	0.66 (0.51–0.85)	0.001	2.399
Fresh vegetables								
Low (0–1)	13	42	31.0	Reference		Reference		
Mod (2–4)	84	226	37.2	1.20 (0.74–1.95)	0.457	0.99 (0.60–1.63)	0.965	1.111
High (5–7)	262	767	34.2	1.10 (0.69–1.75)	0.676	0.85 (0.52–1.39)	0.515	1.632
Red meat								
Low (0–1)	165	469	35.2	Reference		Reference		
Mod (2–4)	188	554	33.9	0.96 (0.81–1.14)	0.676	0.98 (0.80–1.21)	0.855	1.165
High (5–7)	6	12	50.0	1.42 (0.80–2.54)	0.234	1.86 (1.10–3.16)	0.022	3.125
White meat								
Low (0–1)	95	272	34.9	Reference		Reference		
Mod (2–4)	228	659	34.6	0.99 (0.82–1.20)	0.924	0.96 (0.76–1.21)	0.724	1.250
High (5–7)	36	104	34.6	0.99 (0.73–1.35)	0.955	1.03 (0.72–1.46)	0.885	1.206
Fish								
Low (0–1)	233	691	33.7	Reference		Reference		
Mod (2–4)	118	325	36.3	1.08 (0.90–1.29)	0.415	1.02 (0.83–1.27)	0.830	1.163
High (5–7)	8	19	42.1	1.25 (0.73–2.14)	0.418	1.31 (0.70–2.48)	0.400	1.947
Dairy products								
Low (0–1)	72	210	34.3	Reference		Reference		
Mod (2–4)	59	168	35.1	1.02 (0.78–1.35)	0.866	0.91 (0.66–1.26)	0.571	1.429
High (5–7)	228	657	34.7	1.01 (0.82–1.25)	0.912	0.94 (0.71–1.25)	0.675	1.324
Eggs								
Low (0–1)	175	497	35.2	Reference		Reference		
Mod (2–4)	146	433	33.7	0.96 (0.80–1.14)	0.633	0.88 (0.72–1.08)	0.223	1.530
High (5–7)	38	105	36.2	1.03 (0.78–1.36)	0.848	0.89 (0.65–1.21)	0.454	1.496
Soya products								
Low (0–1)	314	896	35.0	Reference		Reference		
Mod (2–4)	33	102	32.4	0.92 (0.69–1.24)	0.595	0.85 (0.60–1.21)	0.372	1.632
High (5–7)	12	37	32.4	0.93 (0.58–1.49)	0.749	0.97 (0.58–1.60)	0.899	1.209
Chocolate								
Low (0–1)	117	356	32.9	Reference		Reference		
Mod (2–4)	168	456	36.8	1.12 (0.93–1.36)	0.241	1.07 (0.86–1.32)	0.545	1.344
High (5–7)	74	223	33.2	1.01 (0.80–1.28)	0.937	1.03 (0.77–1.37)	0.850	1.206
Nuts (almonds or walnuts)								
Low (0–1)	220	613	35.9	Reference		Reference		
Mod (2–4)	92	270	34.1	0.95 (0.78–1.16)	0.605	0.84 (0.68–1.04)	0.106	1.667
High (5–7)	47	152	30.9	0.86 (0.66–1.12)	0.262	0.73 (0.54–0.98)	0.039	2.082

Abbreviations: CI, confidence interval; RR, risk ratio.

^a^ No covariate adjustment.

^b^ Full covariate adjustment (age at conception, BMI, ethnicity, smoking status at baseline, alcohol status at baseline, number of previous live births at baseline, number of previous miscarriages at baseline, linked paternal diet for each specific food group).

^c^ Minimum strength of association an unmeasured confounder would need to have with both the exposure and outcome to explain away the observed effect.

Table S1 shows the sensitivity analyses, demonstrating that both the direction and magnitude of the effect aligned consistently with our primary analyses. Table S2 compares the characteristics of the cohorts included in the primary analyses with those excluded due to missing maternal dietary data.

Data S1 provides the detailed results of our subgroup analyses, confirming the consistency of effects across different populations. Subcategorising the cohort led to a reduced sample size, which produced wider confidence intervals, precluding confident conclusions. While differences between most subgroups remain inconclusive, the analyses centred on maternal age at conception stand out. Notably, in the subgroup analysis based on maternal age at conception, the associations between fresh fruit, red meat and nut consumption seemed markedly more evident in the group with a maternal conception age of 35 years or older. This was due to narrower confidence intervals and larger point estimates in this subgroup, despite both age groups (< 35 and ≥ 35 years) having similar participant numbers.

### 3.2 Overall Dietary Pattern Analysis

We identified two primary dietary patterns characterised by the following components: (1) fresh fruit and vegetables (2) fish and eggs. Table 3 shows the categorisation of the cohort into three groups according to the predictive scoring relating to these dietary patterns, alongside their respective associations with the risk of miscarriage. For the analysis, the first tertile served as the reference group, against which the miscarriage risks of the second and third tertiles were estimated. None of the two identified dietary patterns showed a confident association with miscarriage risk, even after adjustment for maternal confounders. After performing the bootstrapped analysis (Table S4), we observed some adjustments in the point estimates reflecting improved accuracy. However, the confidence intervals remained wide, and thus, the overall conclusions remained consistent, with no clear associations between the data‐derived whole dietary patterns and miscarriage risk.

**TABLE 3:** Poisson regression analysis of overall diet in Tommy's Net recurrent miscarriage cohort by tertiles of data‐derived dietary pattern scores.

Dietary pattern with focus on specified components	Univariable^a^ RR (95% CI)	*p*	Multivariable^b^ RR (95% CI)	*p*
Fresh fruit and vegetables				
T1	Reference		Reference	
T2	0.98 (0.80‐1.21)	0.87	0.96 (0.78‐1.17)	0.66
T3	0.98 (0.79‐1.20)	0.81	0.91 (0.74‐1.13)	0.42
Fish and eggs				
T1	Reference		Reference	
T2	1.01 (0.82‐1.24)	0.96	1.05 (0.85‐1.30)	0.63
T3	1.05 (0.86‐1.29)	0.62	1.11 (0.90‐1.37)	0.33

*Note:* T1: Lowest scoring tertile based on a data‐derived dietary pattern focused on specified components, T2: Middle tertile, T3: Highest scoring tertile.

Abbreviations: CI, confidence interval; RR, risk ratio; T, tertile.

^a^ No covariate adjustment.

^b^ Covariate adjustment for maternal factors: age at conception, BMI, ethnicity, smoking status at baseline, alcohol status at baseline, number of previous live births at baseline, number of previous miscarriages at baseline.

## 4 Discussion

### 4.1 Main Findings

In this prospective cohort study of women with a history of recurrent miscarriages, we observed that higher consumption of fresh fruits, or nuts, specifically almonds and walnuts, was associated with lower miscarriage risk. Conversely, high red meat intake was associated with higher miscarriage risk. However, none of the data‐derived dietary patterns showed a significant association with miscarriage risk.

### 4.2 Strengths and Limitations

The strengths of our study lie in its distinct emphasis on a cohort with a history of recurrent miscarriages. Such a population might be especially attuned to the impacts of diet and lifestyle modifications, as these couples frequently take a proactive approach in seeking clinical advice to mitigate their future miscarriage risk. By targeting this enriched population, our research offers insights into the association between maternal dietary patterns and miscarriage risk. Additionally, by including women with varying definitions of recurrent miscarriage, including biochemical losses, our study benefits from greater generalisability across different recurrent miscarriage populations. While this heterogeneity supports the broader applicability of our findings, we acknowledge that it may have introduced variability, potentially diluting the observed association and making it more challenging to detect precise associations in some food groups. Although detailed subgroup analyses based on miscarriage aetiology were not feasible in our study, future research could benefit from such analyses. Secondly, while much of the prevailing research on diet and miscarriage risk focuses mainly on maternal dietary patterns, our study acknowledges the potential interplay of paternal dietary choices—a covariate rarely available or considered in similar studies. Recognising that pregnancy outcomes are influenced by both maternal and paternal factors, we believe that accounting for paternal diet offers a more comprehensive view of dietary influences on miscarriage risk, representing a significant strength of our study. Further research that considers both maternal and paternal factors will be essential to deepen our understanding of the complex and nuanced relationship between parental dietary choices, embryo quality and subsequent pregnancy outcomes.

The principal limitation of our study is the potential for recall bias, a recurring challenge in nutritional research [31]. To mitigate this, we collected comprehensive baseline data at the initial encounter and used an FFQ, known for its practicality and ability to capture long‐term dietary patterns [32]. While FFQs may lead to under‐ or over‐reporting of certain food items, our FFQ balanced detailed dietary information with feasibility. Additionally, dietary data were collected at entry, with an average interval of 6 months from conception, which may not fully capture changes in diet closer to conception. In the future, cross‐referencing our FFQ data with other diet assessment methods could further strengthen the confidence in our data.

A further limitation to address is the scope of our FFQ. While it efficiently captured the qualitative aspects of diet, it did not delve into specifics regarding quantity. Such FFQs are useful to estimate the intake of particular food items, especially when rooted in an underlying hypothesis. However, their capability to assess the entirety of a diet may be constrained. One of the potential contributing factors for not observing a distinct association between data‐derived whole dietary patterns and miscarriage risk may be tied to the design of our FFQ. Employing a more extensive, quantitative‐focused FFQ could have potentially provided a clearer delineation of this relationship.

Another consideration pertains to the missing data in our dataset which may have led to information bias. We acknowledge that the absence of certain information may not be entirely random, introducing potential selection bias. For example, we were unable to definitively determine whether the missing pregnancy data in some participants were due to a lack of conception or simply a result of loss to follow‐up. This uncertainty prevented us from incorporating conception rates into our analyses, which could have provided valuable context for understanding the relationship between maternal diet and miscarriage risk. Despite these challenges, we employed a multifaceted approach, including multiple imputation models and subgroup analysis with complete dietary information. Neither approach altered our conclusions significantly. Thus, while the missing data and the subsequent sample size might have widened our confidence intervals, our overarching conclusions remain intact.

The observational nature of this study limits our ability to make causal assumptions, as observational studies invariably carry the potential for unmeasured or unknown confounders and biases. Nonetheless, the prospective design of our research is a notable strength, and the consistency observed across various analysis stages strengthens our confidence in our conclusions. The computed E‐values indicate that an unmeasured confounder would need to be associated with both maternal diet and miscarriage risk with a relative risk of 2.399 for fruit, 3.125 for red meat and 2.082 for nuts to fully explain away our findings. While this makes it highly unlikely that a single unmeasured confounder could completely account for the observed associations, the small sample size in the high red meat intake group requires careful consideration. Overall, these findings highlight the potential role of maternal dietary choices in modulating miscarriage risk.

Finally, one important covariate that we were not able to adjust for was socioeconomic status (SES), which is strongly associated with health outcomes. SES is known to shape access to healthy foods, dietary behaviours and broader lifestyle factors, all of which could modulate miscarriage risk. Although SES was a latent variable that could not be directly adjusted for in our analyses, we acknowledge its potential role as a confounder and have illustrated this in our full DAG model. We recognise the need for future studies to integrate this critical variable to provide a more comprehensive understanding of its impact on reproductive outcomes.

### 4.3 Interpretation

A recent systematic review by Chung et al. [23] identified an association between a higher intake of fruits and vegetables and lower odds of miscarriage. Our analysis did not highlight a confident association between moderate to high vegetable intake and lower miscarriage risk. We speculate that the lack of a clear association in our primary analyses might have stemmed from the unique characteristics of our cohort rather than a true absence of an association. It is also notable that the studies included in the meta‐analyses by Chung et al. [23] largely overlooked women who had experienced repeated pregnancy losses—a group whose dietary choices might be significantly influenced by their past experiences. It is plausible that couples in our cohort might have already adjusted their dietary choices in response to previous pregnancy losses. This is supported by our finding that most of our cohort, particularly the maternal participants (fruit: *n* = 662/1035, 64.0% and vegetable: *n* = 767/1035, 74.1%), had a high intake of fresh fruits and vegetables, possibly due to self‐driven dietary and lifestyle changes. As a result, the bulk of our cohort was clustered in the high fresh vegetable consumption category, with a notably smaller subset in the lowest and moderate consumption groups. Therefore, the ability to discern differences in miscarriage risk based on varying consumption amounts is likely to be limited. We note the recurring observation of a lower miscarriage rate with high fruit consumption over that of vegetables. This may be related to differences in nutritional profile, including variations in antioxidant levels [33, 34] and microbiome modulation [35, 36, 37]. Alternatively, higher fruit consumption may serve as a more accurate indicator of a broader range of health‐promoting behaviours.

Our findings on the association between meat consumption and miscarriage risk differed from those of the previous meta‐analysis [23], meriting further attention. Specifically, our study showed an 86% higher miscarriage risk among those with high red meat intake compared to those with low. This disparity should prompt consideration of the specific characteristics of our cohort. It raises the question of whether our findings may be influenced by the unique vulnerabilities of this group, potentially representing an enriched population more susceptible to dietary influences on reproductive outcomes. This hypothesis is supported by a prospective cohort study on a population with subfertility undergoing intracytoplasmic sperm injection cycles, which identified a negative association between red meat consumption and key reproductive markers, such as blastocyst formation, implantation and clinical pregnancy rates [38]. It is plausible that in certain groups, dietary influences may have a more profound effect on the early stages of pregnancy, potentially leading to a range of negative outcomes in the conception and early pregnancy stages. However, careful attention should be drawn that the medium red meat intake group in our study did not show an increased miscarriage risk, with a point estimate close to one and wide confidence intervals overlapping one (188/554 (33.9%) vs. 165/469 (35.2%), RR 0.98, 95% CI 0.80 to 1.21, *p* = 0.855). This may suggest that the relationship between red meat intake and miscarriage risk may not follow a clear linear pattern, where increasing consumption consistently leads to greater risk. It also raises the possibility that the observed association in the high‐intake group may not reflect a true effect, considering the small cohort in the high‐intake group. This warrants further exploration and highlights the need for a more nuanced understanding of dietary impacts in specific populations, especially those with a history of reproductive challenges. While definitive conclusions remain elusive, it is noteworthy that the signal suggesting higher miscarriage risk with high red meat intake aligns with broader evidence on the association between red meat consumption and various pregnancy outcomes. Systematic reviews and meta‐analyses have consistently linked higher red meat intake with increased risk of gestational diabetes [39, 40, 41, 42, 43]. Furthermore, there is evidence suggesting higher risk of preterm labor [44] and hypertensive disorders of pregnancy [44] with higher red meat intake. Beyond pregnancy‐specific contexts, processed red meat is classified as a Group 1 carcinogen by the International Agency for Research on Cancer [45], and high intake is associated with increased risk of cardiovascular disease [46, 47] and type 2 diabetes [48, 49] in the general population. It remains unclear whether these risks are primarily driven by the inherent nutritional composition of red meat or by compounds introduced during processing, cooking or consumption methods, all of which may contribute to adverse health outcomes.

The association between nut consumption and miscarriage risk in our study is noteworthy. Findings from the EARTH study on a population with subfertility did not identify a benefit of total nut intake in reducing the probability of pregnancy loss [50]. However, the impact of nuts on male fertility parameters appears more definitive. A meta‐analysis combining data from two randomised controlled trials demonstrated that men who added nuts to their usual diet experienced significant improvement in sperm motility, vitality and morphology compared to controls [51], as well as a significant reduction in sperm DNA fragmentation in one of the trials [52]. Given that increased sperm DNA fragmentation has been associated with a doubling of miscarriage risk [53], these findings hint at the mechanism by which miscarriage risk might be modulated through higher nut intake among couples.

When examining diet as a whole, we could not delineate a distinct relationship between statistically identified dietary patterns and miscarriage risk. This challenge has also been identified in other studies, especially when assessing diet using an a priori approach and adherence to specific pre‐defined dietary patterns [23, 54, 55]. Evaluating dietary patterns as a whole is essential as we recognise that food groups are not consumed in isolation. Consequently, a choice in one food group inevitably influences choices in others. Yet, it remains challenging to discern the precise relationships between these choices in a way that might yield definitive conclusions. As in our study, dimension‐reducing statistical methods like principal component or factor analysis have been employed to address this issue in nutrition research. However, they still fall short of offering a comprehensive understanding and accurate evaluation of whole dietary patterns and their potential relationship with miscarriage risk.

## 5 Conclusions

In summary, higher maternal consumption of fruits and nuts are associated with a lower miscarriage risk among women with a history of recurrent miscarriage. Further work is needed to confirm these findings and to identify holistic dietary patterns with specific quantities and frequencies of food groups that offer the most benefit.

## Author Contributions

A.D., S.Q. and A.C. contributed to the conception of the study. Y.C. and A.C. contributed to the conception of this work. Y.C., P.M. and S.Q. contributed to the acquisition of data. Y.C., C.E., M.J.P. and A.D. contributed to statistical analyses and generation of tables, figures and Supporting Information. Y.C. has written the first draft of the manuscript with contributions from P.M., C.E., M.J.P., R.D.‐S., S.Q., A.D. and A.C. All co‐authors have provided important intellectual input and interpretation of the results. All authors guarantee that the accuracy and integrity of any part of the work have been appropriately investigated and resolved. All authors have approved the final version of the manuscript. The corresponding author had full access to the data and had final responsibility for the decision to submit it for publication.

## Ethics Statement

This study was approved by West Midlands‐South Birmingham Regional Ethics Committee IRAS No: 213740, 2 225 751 REC Ref: 17/WM/0050: 17/ WM/208.

## Conflicts of Interest

The authors declare no conflicts of interest.

## Supporting information


Data S1.

Data S2.

Figure S1.

Table S1.

Table S2.

Table S3.

Table S4.

## Data Availability

The authors confirm that the data supporting the findings of this study are available within the article and its Supporting Information. Participant‐level personally identifiable data are protected under the UK Data Protection Act 2018 and the General Data Protection Regulation (GDPR) as it applies in the United Kingdom, known as UK GDPR. These laws prohibit the distribution of such data, even in a pseudo‐anonymised form, to ensure the privacy and protection of participants. However, participant‐level data can be made available under a data transfer agreement as part of a collaborative effort, subject to compliance with these legal frameworks. Requests for data sharing will be reviewed by the sponsor (University of Birmingham) under the ethical approval granted by the West Midlands Research Ethics Committee, ensuring all data sharing adheres to ethical and legal standards.
