# Supplementary material for: The Association Between Periconceptual Maternal Dietary Patterns and Miscarriage Risk in Women With Recurrent Miscarriages: A Multicentre Cohort Study
Source: BJOG. 2024 Nov 26;132(4):504–17. doi: 10.1111/1471-0528.18022 (PMC11794061; doi:10.1111/1471-0528.18022)
Supplement: Supplementary file 1 — Data S1. [file BJO-132-504-s003.pdf]

Material S1. Table 1. Subgroup analysis for maternal age at conception with covariate adjustment*

Food items (days per week)	Maternal age <35 years		Maternal age ≥35 years	
	RR (95% CI)	p-value	RR (95% CI)	p-value
Total number	442		427	
**Fresh fruit**				
low (0-1)	reference		reference	
mod (2-4)	0.67 (0.45 - 1.02)	0.061	0.51 (0.34 - 0.77)	0.001
high (5-7)	0.69 (0.46 - 1.03)	0.068	0.60 (0.43 - 0.84)	0.002
**Fresh vegetables**				
low (0-1)	reference		reference	
mod (2-4)	1.16 (0.53 - 2.55)	0.706	0.78 (0.40 - 1.51)	0.465
high (5-7)	0.89 (0.40 - 2.00)	0.778	0.78 (0.41 - 1.46)	0.433
**Red meat**				
low (0-1)	reference		reference	
mod (2-4)	0.87 (0.64 - 1.19)	0.397	1.09 (0.83 - 1.43)	0.548
high (5-7)	1.38 (0.73 - 2.58)	0.319	3.21 (1.79 - 5.75)	0.000
**White meat**				
low (0-1)	reference		reference	
mod (2-4)	1.17 (0.78 - 1.77)	0.444	0.82 (0.62 - 1.08)	0.155
high (5-7)	0.98 (0.53 - 1.80)	0.942	1.17 (0.76 - 1.79)	0.478
**Fish**				
low (0-1)	reference		reference	
mod (2-4)	1.18 (0.86 - 1.61)	0.313	0.94 (0.71 - 1.25)	0.672
high (5-7)	1.22 (0.46 - 3.27)	0.693	1.30 (0.56 - 3.03)	0.536
**Dairy products**				
low (0-1)	reference		reference	
mod (2-4)	1.03 (0.60 - 1.76)	0.918	0.84 (0.55 - 1.26)	0.391
high (5-7)	0.97 (0.60 - 1.56)	0.886	0.91 (0.64 - 1.30)	0.609
**Eggs**				
low (0-1)	reference		reference	
mod (2-4)	0.96 (0.71 - 1.29)	0.777	0.85 (0.65 - 1.11)	0.243
high (5-7)	0.67 (0.36 - 1.24)	0.200	1.05 (0.73 - 1.53)	0.781
**Soya products**				
low (0-1)	reference		reference	
mod (2-4)	1.02 (0.59 - 1.77)	0.941	0.76 (0.49 - 1.20)	0.241
high (5-7)	0.83 (0.36 - 1.89)	0.651	1.12 (0.60 - 2.10)	0.723
**Chocolate**				
low (0-1)	reference		reference	
mod (2-4)	1.30 (0.91 - 1.85)	0.146	0.91 (0.69 - 1.19)	0.483
high (5-7)	1.35 (0.88 - 2.07)	0.175	0.83 (0.56 - 1.23)	0.353
**Nuts (almonds or walnuts)**				
low (0-1)	reference		reference	
mod (2-4)	0.96 (0.67 - 1.37)	0.818	0.76 (0.57 - 1.00)	0.048
high (5-7)	0.79 (0.48 - 1.32)	0.373	0.72 (0.49 - 1.05)	0.091

Material S1. Table 2. Subgroup analysis for maternal BMI with covariate adjustment*

Food items (days per week)	BMI < 19		BMI 19 - 25		BMI ≥ 25	
	RR (95% CI)	p-value	RR (95% CI)	*p-value*	RR (95% CI)	*p-value*
Total number	20		397		452	
**Fresh fruit**						
low (0-1)	Insufficient numbers for analysis		reference		reference	
mod (2-4)			0.69 (0.45 - 1.07)	*0.099*	0.55 (0.38 - 0.81)	*0.002*
high (5-7)			0.58 (0.39 - 0.87)	*0.009*	0.70 (0.50 - 0.97)	*0.034*
**Fresh vegetables**						
low (0-1)	Insufficient numbers for analysis		reference		reference	
mod (2-4)			0.68 (0.29 - 1.61)	*0.383*	0.99 (0.54 - 1.82)	*0.975*
high (5-7)			0.51 (0.21 - 1.22)	*0.132*	0.97 (0.53 - 1.76)	*0.912*
**Red meat**						
low (0-1)	Insufficient numbers for analysis		reference		reference	
mod (2-4)			0.94 (0.69 - 1.28)	*0.690*	1.07 (0.81 - 1.42)	*0.627*
high (5-7)			2.24 (1.39 - 3.60)	*0.001*	1.69 (0.69 - 4.17)	*0.253*
**White meat**						
low (0-1)	Insufficient numbers for analysis		reference		reference	
mod (2-4)			1.03 (0.74 - 1.43)	*0.879*	0.91 (0.67 - 1.25)	*0.564*
high (5-7)			0.83 (0.46 - 1.51)	*0.545*	1.07 (0.68 - 1.68)	*0.783*
**Fish**						
low (0-1)	Insufficient numbers for analysis		reference		reference	
mod (2-4)			0.82 (0.59 - 1.14)	*0.232*	1.23 (0.93 - 1.62)	*0.147*
high (5-7)			1.20 (0.37 - 3.86)	*0.764*	1.71 (0.81 - 3.59)	*0.156*
**Dairy products**						
low (0-1)	Insufficient numbers for analysis		reference		reference	
mod (2-4)			0.79 (0.46 - 1.33)	*0.369*	1.01 (0.67 - 1.52)	*0.958*
high (5-7)			0.97 (0.64 - 1.47)	*0.888*	0.94 (0.64 - 1.37)	*0.733*
**Eggs**						
low (0-1)	Insufficient numbers for analysis		reference		reference	
mod (2-4)			0.82 (0.60 - 1.12)	*0.203*	0.94 (0.73 - 1.22)	*0.665*
high (5-7)			0.70 (0.38 - 1.28)	*0.245*	1.06 (0.74 - 1.54)	*0.740*
**Soya products**						
low (0-1)	Insufficient numbers for analysis		reference		reference	
mod (2-4)			0.78 (0.46 - 1.34)	*0.376*	1.00 (0.64 - 1.58)	*0.987*
high (5-7)			0.92 (0.40 - 2.13)	*0.845*	1.02 (0.56 - 1.86)	*0.945*
**Chocolate**						
low (0-1)	Insufficient numbers for analysis		reference		reference	
mod (2-4)			1.27 (0.90 - 1.77)	*0.171*	0.98 (0.75 - 1.28)	*0.867*
high (5-7)			1.33 (0.87 - 2.03)	*0.185*	0.86 (0.58 - 1.26)	*0.431*
**Nuts (almonds or walnuts)**						
low (0-1)	Insufficient numbers for analysis		reference		reference	
mod (2-4)			0.85 (0.61 - 1.19)	*0.338*	0.81 (0.61 - 1.07)	*0.131*
high (5-7)			0.73 (0.48 - 1.12)	*0.148*	0.76 (0.49 - 1.18)	*0.224*

Material S1. Table 3. Subgroup analysis for maternal ethnicity with covariate adjustment*

Food items (days per week)	White		mixed		asian		black		other	
	RR (95% CI)	*p-value*	RR (95% CI)	*p-value*	RR (95% CI)	*p-value*	RR (95% CI)	*p-value*	RR (95% CI)	*p-value*
Total number	757		14		66		16		16	
**Fresh fruit**										
low (0-1)	reference		Insufficient numbers for analysis		reference		Insufficient numbers for analysis		Insufficient numbers for analysis	
mod (2-4)	0.64 (0.47 - 0.88)	*0.005*			0.81 (0.30 - 2.16)	*0.668*				
high (5-7)	0.65 (0.49 - 0.86)	*0.002*			0.85 (0.36 - 2.00)	*0.703*				
**Fresh vegetables**										
low (0-1)	reference		Insufficient numbers for analysis		reference		Insufficient numbers for analysis		Insufficient numbers for analysis	
mod (2-4)	1.04 (0.59 - 1.82)	*0.887*			0.85 (0.27 - 2.73)	*0.791*				
high (5-7)	0.83 (0.47 - 1.46)	*0.519*			0.80 (0.27 - 2.34)	*0.685*				
**Red meat**										
low (0-1)	reference		Insufficient numbers for analysis		reference		Insufficient numbers for analysis		Insufficient numbers for analysis	
mod (2-4)	1.02 (0.82 - 1.27)	*0.852*			2.13 (1.00 - 4.56)	*0.051*				
high (5-7)	1.84 (0.97 - 3.47)	*0.061*			1.70 (0.54 - 5.36)	*0.364*				
**White meat**										
low (0-1)	reference		Insufficient numbers for analysis		reference		Insufficient numbers for analysis		Insufficient numbers for analysis	
mod (2-4)	0.89 (0.60 - 1.33)	*0.574*			0.65 (0.21 - 2.02)	*0.458*				
high (5-7)	0.78 (0.37 - 1.61)	*0.496*			0.39 (0.04 - 3.61)	*0.405*				
**Fish**										
low (0-1)	reference		Insufficient numbers for analysis		reference		Insufficient numbers for analysis		Insufficient numbers for analysis	
mod (2-4)	0.99 (0.79 - 1.25)	*0.931*			1.89 (1.00 - 3.58)	*0.051*				
high (5-7)	1.07 (0.46 - 2.48)	*0.88*			3.50 (0.89 - 13.86)	*0.074*				
**Dairy products**										
low (0-1)	reference		Insufficient numbers for analysis		reference		Insufficient numbers for analysis		Insufficient numbers for analysis	
mod (2-4)	0.92 (0.66 - 1.29)	*0.636*			0.81 (0.30 - 2.18)	*0.68*				
high (5-7)	0.84 (0.63 - 1.13)	*0.257*			0.83 (0.31 - 2.21)	*0.704*				
**Eggs**										
low (0-1)	reference		Insufficient numbers for analysis		reference		Insufficient numbers for analysis		Insufficient numbers for analysis	
mod (2-4)	0.83 (0.67 - 1.03)	*0.093*			1.37 (0.73 - 2.55)	*0.328*				
high (5-7)	0.91 (0.65 - 1.27)	*0.573*			0.76 (0.23 - 2.54)	*0.653*				
**Soya products**										
low (0-1)	reference		Insufficient numbers for analysis		reference		Insufficient numbers for analysis		Insufficient numbers for analysis	
mod (2-4)	0.83 (0.57 - 1.22)	*0.346*			0.29 (0.06 - 1.36)	*0.117*				
high (5-7)	0.85 (0.47 - 1.53)	*0.586*			0.71 (0.11 - 4.67)	*0.722*				
**Chocolate**										
low (0-1)	reference		Insufficient numbers for analysis		reference		Insufficient numbers for analysis		Insufficient numbers for analysis	
mod (2-4)	1.06 (0.84 - 1.33)	*0.623*			1.53 (0.74 - 3.14)	*0.249*				
high (5-7)	0.96 (0.70 - 1.31)	*0.788*			2.35 (1.19 - 4.62)	*0.014*				
**Nuts (almonds or walnuts)**										
low (0-1)	reference		Insufficient numbers for analysis		reference		Insufficient numbers for analysis		Insufficient numbers for analysis	
mod (2-4)	0.87 (0.69 - 1.10)	*0.235*			0.59 (0.31 - 1.12)	*0.109*				
high (5-7)	0.76 (0.55 - 1.05)	*0.095*			0.34 (0.13 - 0.87)	*0.024*				

Material S1. Table 4. Subgroup analysis for baseline live birth history with covariate adjustment*

Food items (days per week)	No baseline live birth		Yes baseline live birth	
	RR (95% CI)	*p-value*	RR (95% CI)	*p-value*
Total number	559		310	
**Fresh fruit**				
low (0-1)	reference		reference	
mod (2-4)	0.60 (0.43 - 0.84)	*0.002*	0.69 (0.37 - 1.30)	*0.254*
high (5-7)	0.59 (0.44 - 0.79)	*0.000*	0.86 (0.48 - 1.54)	*0.603*
**Fresh vegetables**				
low (0-1)	reference		reference	
mod (2-4)	0.72 (0.44 - 1.17)	*0.187*	3.45 (0.49 - 24.37)	*0.214*
high (5-7)	0.60 (0.37 - 0.96)	*0.032*	3.27 (0.45 - 23.94)	*0.243*
**Red meat**				
low (0-1)	reference		reference	
mod (2-4)	0.96 (0.75 - 1.22)	*0.732*	1.09 (0.74 - 1.61)	*0.664*
high (5-7)	1.38 (0.66 - 2.90)	*0.388*	3.48 (1.35 - 8.98)	*0.010*
**White meat**				
low (0-1)	reference		reference	
mod (2-4)	0.91 (0.69 - 1.20)	*0.508*	1.05 (0.69 - 1.61)	*0.813*
high (5-7)	0.92 (0.60 - 1.39)	*0.683*	1.29 (0.68 - 2.46)	*0.440*
**Fish**				
low (0-1)	reference		reference	
mod (2-4)	1.03 (0.80 - 1.34)	*0.801*	1.00 (0.68 - 1.48)	*0.990*
high (5-7)	1.25 (0.47 - 3.30)	*0.656*	1.24 (0.57 - 2.70)	*0.594*
**Dairy products**				
low (0-1)	reference		reference	
mod (2-4)	0.89 (0.63 - 1.27)	*0.533*	0.86 (0.40 - 1.83)	*0.691*
high (5-7)	0.86 (0.62 - 1.18)	*0.347*	1.15 (0.64 - 2.06)	*0.643*
**Eggs**				
low (0-1)	reference		reference	
mod (2-4)	0.90 (0.71 - 1.15)	*0.395*	0.85 (0.59 - 1.23)	*0.399*
high (5-7)	0.85 (0.58 - 1.23)	*0.385*	0.94 (0.52 - 1.67)	*0.827*
**Soya products**				
low (0-1)	reference		reference	
mod (2-4)	0.90 (0.59 - 1.39)	*0.647*	0.78 (0.43 - 1.41)	*0.407*
high (5-7)	1.00 (0.56 - 1.77)	*0.999*	0.95 (0.38 - 2.37)	*0.906*
**Chocolate**				
low (0-1)	reference		reference	
mod (2-4)	1.02 (0.79 - 1.32)	*0.867*	1.22 (0.83 - 1.79)	*0.320*
high (5-7)	1.10 (0.78 - 1.54)	*0.599*	0.94 (0.57 - 1.53)	*0.799*
**Nuts (almonds or walnuts)**				
low (0-1)	reference		reference	
mod (2-4)	0.80 (0.62 - 1.04)	*0.094*	0.93 (0.63 - 1.37)	*0.728*
high (5-7)	0.79 (0.56 - 1.12)	*0.184*	0.58 (0.31 - 1.09)	*0.093*

Material S1. Table 5. Subgroup analysis for baseline miscarriage history with covariate adjustment*

Food items (days per week)	baseline misc = 2		baseline misc = 3		baseline misc = 4		baseline misc ≤5	
	RR (95% CI)	*p-value*	RR (95% CI)	*p-value*	RR (95% CI)	*p-value*	RR (95% CI)	*p-value*
Total number	271		282		154		77	
**Fresh fruit**								
low (0-1)	reference		reference		reference		reference	
mod (2-4)	0.45 (0.26 - 0.78)	*0.004*	0.98 (0.47 - 2.05)	*0.960*	0.53 (0.26 - 1.05)	*0.070*	0.54 (0.19 - 1.53)	*0.245*
high (5-7)	0.48 (0.30 - 0.75)	*0.001*	0.94 (0.46 - 1.93)	*0.865*	0.76 (0.42 - 1.39)	*0.376*	0.59 (0.24 - 1.48)	*0.259*
**Fresh vegetables**								
low (0-1)	reference		reference		reference		reference	
mod (2-4)	0.61 (0.32 - 1.16)	*0.132*	0.89 (0.37 - 2.10)	*0.783*	1.84 (0.37 - 9.21)	*0.456*	0.73 (0.15 - 3.62)	*0.702*
high (5-7)	0.54 (0.30 - 1.00)	*0.049*	0.70 (0.28 - 1.73)	*0.435*	1.70 (0.35 - 8.28)	*0.511*	0.87 (0.20 - 3.77)	*0.856*
**Red meat**								
low (0-1)	reference				reference			
mod (2-4)	1.05 (0.67 - 1.64)	*0.825*	Insufficient numbers for analysis		1.00 (0.66 - 1.52)	*0.996*	Insufficient numbers for analysis	
high (5-7)	2.48 (0.50 - 12.34)	*0.266*			3.15 (1.35 - 7.36)	*0.008*		
**White meat**								
low (0-1)	reference		reference		reference		reference	
mod (2-4)	0.95 (0.58 - 1.54)	*0.825*	0.86 (0.58 - 1.28)	*0.462*	1.00 (0.61 - 1.62)	*0.993*	1.09 (0.55 - 2.17)	*0.801*
high (5-7)	0.83 (0.35 - 2.01)	*0.683*	0.68 (0.34 - 1.34)	*0.261*	1.24 (0.64 - 2.43)	*0.526*	1.38 (0.52 - 3.66)	*0.513*
**Fish**								
low (0-1)	reference		reference		reference			
mod (2-4)	1.26 (0.79 - 2.00)	*0.334*	0.91 (0.60 - 1.38)	*0.663*	1.19 (0.81 - 1.76)	*0.373*	Insufficient numbers for analysis	
high (5-7)	1.85 (0.66 - 5.17)	*0.239*	0.95 (0.30 - 3.03)	*0.938*	0.89 (0.36 - 2.20)	*0.800*		
**Dairy products**								
low (0-1)	reference		reference		reference		reference	
mod (2-4)	0.80 (0.38 - 1.67)	*0.546*	0.91 (0.46 - 1.82)	*0.791*	1.02 (0.52 - 2.00)	*0.954*	1.84 (0.74 - 4.56)	*0.189*
high (5-7)	0.77 (0.41 - 1.46)	*0.429*	1.12 (0.63 - 1.98)	*0.710*	0.93 (0.49 - 1.75)	*0.812*	1.35 (0.54 - 3.33)	*0.519*
**Eggs**								
low (0-1)	reference		reference		reference		reference	
mod (2-4)	1.03 (0.67 - 1.60)	*0.883*	1.00 (0.69 - 1.45)	*0.999*	0.59 (0.37 - 0.93)	*0.023*	0.63 (0.35 - 1.14)	*0.126*
high (5-7)	1.05 (0.40 - 2.73)	*0.919*	0.81 (0.44 - 1.48)	*0.488*	0.72 (0.40 - 1.29)	*0.274*	0.90 (0.43 - 1.88)	*0.785*
**Soya products**								
low (0-1)	reference		reference		reference		reference	
mod (2-4)	0.65 (0.41 - 1.04)	*0.075*	0.83 (0.47 - 1.47)	*0.527*	0.89 (0.49 - 1.62)	*0.699*	0.74 (0.28 - 1.97)	*0.541*
high (5-7)	0.68 (0.35 - 1.34)	*0.264*	0.35 (0.06 - 1.98)	*0.233*	0.94 (0.45 - 1.97)	*0.877*	1.04 (0.50 - 2.16)	*0.912*
**Chocolate**								
low (0-1)	reference		reference		reference		reference	
mod (2-4)	1.14 (0.46 - 2.79)	*0.781*	1.35 (0.88 - 2.06)	*0.168*	1.36 (0.85 - 2.19)	*0.202*	1.29 (0.76 - 2.20)	*0.345*
high (5-7)	1.34 (0.51 - 3.49)	*0.551*	1.39 (0.84 - 2.32)	*0.204*	1.54 (0.88 - 2.69)	*0.128*	0.45 (0.13 - 1.49)	*0.190*
**Nuts (almonds or walnuts)**								
low (0-1)	reference		reference		reference		reference	
mod (2-4)	0.70 (0.39 - 1.24)	*0.217*	1.06 (0.74 - 1.54)	*0.741*	0.80 (0.49 - 1.31)	*0.373*	0.42 (0.20 - 0.88)	*0.021*
high (5-7)	0.69 (0.36 - 1.34)	*0.274*	0.62 (0.35 - 1.09)	*0.096*	0.77 (0.41 - 1.42)	*0.401*	0.69 (0.36 - 1.35)	*0.284*

Material S1. Table 6. Subgroup analysis for baseline alcohol status with covariate adjustment*

Food items (days per week)	No alcohol		Yes alcohol	
	RR (95% CI)	*p-value*	RR (95% CI)	*p-value*
Total number	405		464	
**Fresh fruit**				
low (0-1)	reference		reference	
mod (2-4)	0.76 (0.47 - 1.23)	*0.27*	0.49 (0.34 - 0.71)	*0.00*
high (5-7)	0.88 (0.57 - 1.37)	*0.58*	0.50 (0.36 - 0.68)	*0.00*
**Fresh vegetables**				
low (0-1)	reference		reference	
mod (2-4)	1.26 (0.58 - 2.75)	*0.56*	0.77 (0.40 - 1.47)	*0.43*
high (5-7)	1.19 (0.54 - 2.60)	*0.67*	0.59 (0.31 - 1.12)	*0.11*
**Red meat**				
low (0-1)	reference		reference	
mod (2-4)	1.03 (0.75 - 1.42)	*0.84*	0.95 (0.72 - 1.25)	*0.72*
high (5-7)	2.10 (1.02 - 4.29)	*0.04*	1.64 (0.75 - 3.57)	*0.22*
**White meat**				
low (0-1)	reference		reference	
mod (2-4)	0.94 (0.66 - 1.32)	*0.71*	0.97 (0.71 - 1.31)	*0.84*
high (5-7)	0.76 (0.44 - 1.34)	*0.35*	1.25 (0.80 - 1.95)	*0.32*
**Fish**				
low (0-1)	reference		reference	
mod (2-4)	1.05 (0.76 - 1.45)	*0.77*	1.00 (0.74 - 1.34)	*0.99*
high (5-7)	2.44 (1.20 - 4.97)	*0.01*	0.67 (0.23 - 1.97)	*0.46*
**Dairy products**				
low (0-1)	reference		reference	
mod (2-4)	0.92 (0.57 - 1.48)	*0.72*	0.86 (0.56 - 1.31)	*0.48*
high (5-7)	0.96 (0.63 - 1.46)	*0.84*	0.90 (0.62 - 1.31)	*0.59*
**Eggs**				
low (0-1)	reference		reference	
mod (2-4)	1.02 (0.76 - 1.37)	*0.89*	0.79 (0.60 - 1.04)	*0.09*
high (5-7)	0.81 (0.49 - 1.34)	*0.41*	0.94 (0.63 - 1.41)	*0.77*
**Soya products**				
low (0-1)	reference		reference	
mod (2-4)	0.65 (0.33 - 1.26)	*0.20*	1.04 (0.70 - 1.54)	*0.85*
high (5-7)	1.22 (0.64 - 2.30)	*0.55*	0.83 (0.39 - 1.78)	*0.64*
**Chocolate**				
low (0-1)	reference		reference	
mod (2-4)	0.92 (0.67 - 1.26)	*0.59*	1.19 (0.89 - 1.58)	*0.25*
high (5-7)	1.08 (0.73 - 1.58)	*0.70*	0.98 (0.65 - 1.49)	*0.94*
**Nuts (almonds or walnuts)**				
low (0-1)	reference		reference	
mod (2-4)	0.82 (0.60 - 1.14)	*0.24*	0.84 (0.63 - 1.12)	*0.24*
high (5-7)	0.79 (0.50 - 1.23)	*0.29*	0.68 (0.46 - 1.01)	*0.06*

Material S1. Table 7. Subgroup analysis for baseline smoking status with covariate adjustment*

Food items (days per week)	No smoking at baseline		Yes smoking at baseline	
	RR (95% CI)	*p-value*	RR (95% CI)	*p-value*
Total number	819		50	
**Fresh fruit**				
low (0-1)	reference		reference	
mod (2-4)	0.60 (0.44 - 0.81)	*0.001*	0.51 (0.18 - 1.46)	*0.213*
high (5-7)	0.66 (0.50 - 0.85)	*0.002*	0.50 (0.13 - 2.01)	*0.331*
**Fresh vegetables**				
low (0-1)	reference		reference	
mod (2-4)	0.92 (0.55 - 1.55)	*0.768*	1.38 (0.20 - 9.67)	*0.747*
high (5-7)	0.82 (0.49 - 1.38)	*0.459*	1.00 (0.13 - 7.63)	*0.998*
**Red meat**				
low (0-1)	reference			
mod (2-4)	1.01 (0.81 - 1.25)	*0.944*	Insufficient numbers for analysis	
high (5-7)	2.13 (1.32 - 3.42)	*0.002*		
**White meat**				
low (0-1)	reference		reference	
mod (2-4)	0.98 (0.77 - 1.24)	*0.856*	0.51 (0.19 - 1.38)	*0.185*
high (5-7)	1.05 (0.73 - 1.52)	*0.778*	0.35 (0.08 - 1.49)	*0.155*
**Fish**				
low (0-1)	reference			
mod (2-4)	1.01 (0.81 - 1.26)	*0.916*	Insufficient numbers for analysis	
high (5-7)	1.37 (0.73 - 2.58)	*0.323*		
**Dairy products**				
low (0-1)	reference		reference	
mod (2-4)	0.93 (0.67 - 1.30)	*0.690*	0.42 (0.09 - 1.98)	*0.275*
high (5-7)	0.98 (0.73 - 1.31)	*0.875*	0.44 (0.10 - 1.94)	*0.278*
**Eggs**				
low (0-1)	reference		reference	
mod (2-4)	0.89 (0.73 - 1.10)	*0.282*	0.93 (0.42 - 2.08)	*0.866*
high (5-7)	0.91 (0.67 - 1.25)	*0.578*	0.51 (0.11 - 2.34)	*0.384*
**Soya products**				
low (0-1)	reference		reference	
mod (2-4)	0.81 (0.56 - 1.18)	*0.266*	1.23 (0.25 - 6.09)	*0.803*
high (5-7)	0.88 (0.51 - 1.51)	*0.636*	6.00 (1.56 - 23.08)	*0.009*
**Chocolate**				
low (0-1)	reference		reference	
mod (2-4)	1.14 (0.91 - 1.42)	*0.258*	0.48 (0.21 - 1.10)	*0.084*
high (5-7)	1.09 (0.82 - 1.46)	*0.547*	0.50 (0.11 - 2.31)	*0.376*
**Nuts (almonds or walnuts)**				
low (0-1)	reference		reference	
mod (2-4)	0.85 (0.68 - 1.05)	*0.133*	0.74 (0.21 - 2.62)	*0.639*
high (5-7)	0.71 (0.52 - 0.97)	*0.030*	2.74 (1.18 - 6.35)	*0.019*

*covariate adjustment for maternal factors and paternal diet pattern: age at conception, BMI, ethnicity, smoking status at baseline, alcohol status at baseline, number of previous live births at baseline, number of previous miscarriages at baseline, linked paternal diet for each specific food group
