# Supplementary figures and images for: The Association Between Periconceptual Maternal Dietary Patterns and Miscarriage Risk in Women With Recurrent Miscarriages: A Multicentre Cohort Study
Source: BJOG. 2024 Nov 26;132(4):504–17. doi: 10.1111/1471-0528.18022 (PMC11794061; doi:10.1111/1471-0528.18022)

**Material S2.** Tommy’s Net Food Frequency Questionnaire


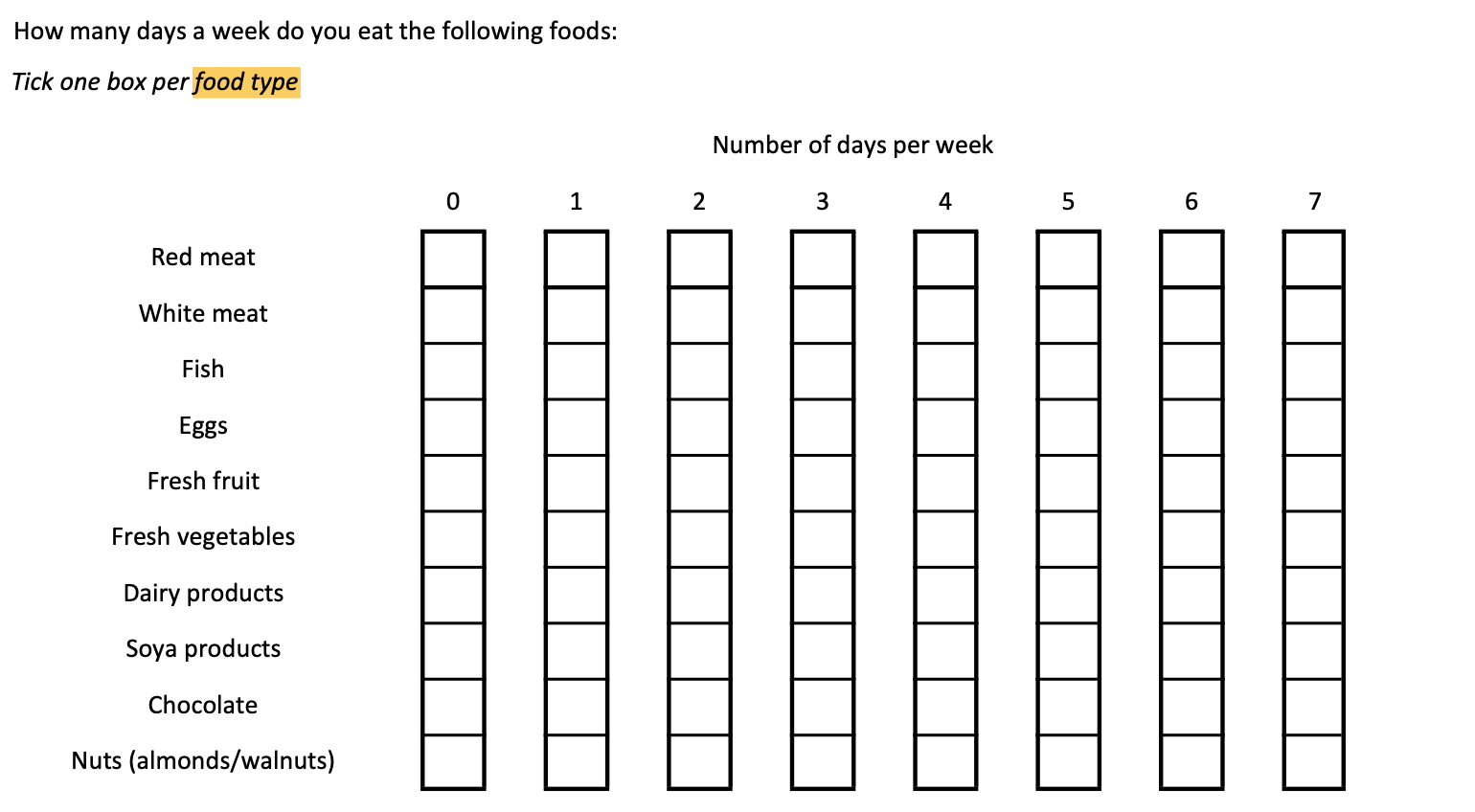

Supplement: Supplementary file 2 — Data S2. [file BJO-132-504-s002.docx]
