# Supplementary material for: The Association Between Periconceptual Maternal Dietary Patterns and Miscarriage Risk in Women With Recurrent Miscarriages: A Multicentre Cohort Study
Source: BJOG. 2024 Nov 26;132(4):504–17. doi: 10.1111/1471-0528.18022 (PMC11794061; doi:10.1111/1471-0528.18022)
Supplement: Supplementary file 3 — Figure S1. [file BJO-132-504-s005.docx]

### Figures S1

| 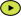 | Exposure |
| --- | --- |
| 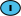 | Outcome |
| 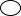 | Adjusted variable |
| 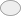 | Unobserved (latent) variable |
| 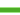 | Causal path |
|  |  |

**Footnotes**

The full version of the DAG can be accessed at <https://dagitty.net/mTFWRU6e2>.
