# Supplementary material for: The Association Between Periconceptual Maternal Dietary Patterns and Miscarriage Risk in Women With Recurrent Miscarriages: A Multicentre Cohort Study
Source: BJOG. 2024 Nov 26;132(4):504–17. doi: 10.1111/1471-0528.18022 (PMC11794061; doi:10.1111/1471-0528.18022)
Supplement: Supplementary file 4 — Table S1. [file BJO-132-504-s001.docx]

**Table S1.** Sensitivity analysis using Poisson regression of individual food categories in Tommy’s Net recurrent miscarriage cohort based on Complete maternal and paternal dietary data and Multiple Imputation

| **Food items**  (days per week) |  | **Miscarriage number** |  | **Total**  **pregnancy number** |  | **Miscarriage**  **Rate** (%) |  | **Univariable^1^** | |  | **Complete Case Multivariable^2^** | |  | **Multiple Imputation Multivariable^3^**  *(Missing covariates)* | |  | **Multiple Imputation**  **Multivariable^4^**  *(Missing maternal diet)* | |
| --- | --- | --- | --- | --- | --- | --- | --- | --- | --- | --- | --- | --- | --- | --- | --- | --- | --- | --- |
|  |  |  |  |  |  |  |  | RR (95% CI) | p-value |  | RR (95% CI) | p-value |  | RR (95% CI) | p-value |  | RR (95% CI) | p-value |
|  |  |  |  |  |  |  |  |  |  |  |  |  |  |  |  |  |  |  |
| **Fresh fruit** |  |  |  |  |  |  |  |  |  |  |  |  |  |  |  |  |  |  |
| low (0-1) |  | 32 |  | 60 |  | 53.3 |  | reference |  |  | reference |  |  | reference |  |  | reference |  |
| mod (2-4) |  | 81 |  | 256 |  | 31.6 |  | 0.59 (0.44 - 0.80) | 0.001 |  | 0.61 (0.46 - 0.82) | 0.001 |  | 0.68 (0.52 - 0.89) | 0.005 |  | 0.76 (0.58 - 0.98) | 0.035 |
| high (5-7) |  | 281 |  | 653 |  | 43.0 |  | 0.66 (0.51 - 0.85) | 0.002 |  | 0.66 (0.51 - 0.85) | 0.001 |  | 0.69 (0.54 - 0.88) | 0.003 |  | 0.74 (0.57 - 0.95) | 0.021 |
|  |  |  |  |  |  |  |  |  |  |  |  |  |  |  |  |  |  |  |
| **Fresh vegetables** |  |  |  |  |  |  |  |  |  |  |  |  |  |  |  |  |  |  |
| low (0-1) |  | 11 |  | 32 |  | 34.4 |  | reference |  |  | reference |  |  | reference |  |  | reference |  |
| mod (2-4) |  | 71 |  | 192 |  | 37.0 |  | 1.08 (0.64 - 1.80) | 0.780 |  | 0.99 (0.60 - 1.63) | 0.965 |  | 1.04 (0.65 - 1.66) | 0.868 |  | 1.01 (0.68 - 1.49) | 0.978 |
| high (5-7) |  | 232 |  | 665 |  | 34.9 |  | 1.01 (0.62 - 1.66) | 0.953 |  | 0.85 (0.52 - 1.39) | 0.515 |  | 0.87 (0.54 - 1.39) | 0.553 |  | 0.84 (0.56 - 1.28) | 0.417 |
|  |  |  |  |  |  |  |  |  |  |  |  |  |  |  |  |  |  |  |
| **Red meat** |  |  |  |  |  |  |  |  |  |  |  |  |  |  |  |  |  |  |
| low (0-1) |  | 138 |  | 398 |  | 34.7 |  | reference |  |  | reference |  |  | reference |  |  | reference |  |
| mod (2-4) |  | 170 |  | 481 |  | 35.3 |  | 1.02 (0.85 - 1.22) | 0.836 |  | 0.98 (0.80 - 1.21) | 0.855 |  | 0.93 (0.77 - 1.13) | 0.470 |  | 0.94 (0.77 - 1.14) | 0.522 |
| high (5-7) |  | 6 |  | 10 |  | 60.0 |  | 1.73 (1.02 - 2.92) | 0.040 |  | 1.86 (1.10 - 3.16) | 0.022 |  | 1.55 (0.88 - 2.73) | 0.129 |  | 1.35 (0.71 - 2.56) | 0.360 |
|  |  |  |  |  |  |  |  |  |  |  |  |  |  |  |  |  |  |  |
| **White meat** |  |  |  |  |  |  |  |  |  |  |  |  |  |  |  |  |  |  |
| low (0-1) |  | 82 |  | 225 |  | 36.4 |  | reference |  |  | reference |  |  | reference |  |  | reference |  |
| mod (2-4) |  | 198 |  | 571 |  | 34.7 |  | 0.95 (0.77 - 1.17) | 0.636 |  | 0.96 (0.76 - 1.21) | 0.724 |  | 1.00 (0.81 - 1.24) | 0.972 |  | 0.94 (0.78 - 1.14) | 0.554 |
| high (5-7) |  | 34 |  | 93 |  | 36.6 |  | 1.00 (0.73 - 1.38) | 0.985 |  | 1.03 (0.72 - 1.46) | 0.885 |  | 0.98 (0.69 - 1.38) | 0.890 |  | 0.90 (0.65 - 1.25) | 0.521 |
|  |  |  |  |  |  |  |  |  |  |  |  |  |  |  |  |  |  |  |
| **Fish** |  |  |  |  |  |  |  |  |  |  |  |  |  |  |  |  |  |  |
| low (0-1) |  | 208 |  | 600 |  | 34.7 |  | reference |  |  | reference |  |  | reference |  |  | reference |  |
| mod (2-4) |  | 100 |  | 273 |  | 36.6 |  | 1.06 (0.87 - 1.28) | 0.572 |  | 1.02 (0.83 - 1.27) | 0.830 |  | 1.04 (0.85 - 1.27) | 0.697 |  | 1.06 (0.87 - 1.30) | 0.545 |
| high (5-7) |  | 6 |  | 16 |  | 37.5 |  | 1.08 (0.57 - 2.06) | 0.811 |  | 1.31 (0.70 - 2.48) | 0.400 |  | 1.29 (0.73 - 2.29) | 0.387 |  | 1.42 (0.82 - 2.44) | 0.207 |
|  |  |  |  |  |  |  |  |  |  |  |  |  |  |  |  |  |  |  |
| **Dairy products** |  |  |  |  |  |  |  |  |  |  |  |  |  |  |  |  |  |  |
| low (0-1) |  | 63 |  | 176 |  | 35.8 |  | reference |  |  | reference |  |  | reference |  |  | reference |  |
| mod (2-4) |  | 48 |  | 140 |  | 34.3 |  | 0.96 (0.71 - 1.30) | 0.780 |  | 0.91 (0.66 - 1.26) | 0.571 |  | 0.95 (0.70 - 1.27) | 0.711 |  | 0.94 (0.72 - 1.21) | 0.620 |
| high (5-7) |  | 203 |  | 573 |  | 35.4 |  | 0.99 (0.79 - 1.24) | 0.929 |  | 0.94 (0.71 - 1.25) | 0.675 |  | 0.95 (0.72 - 1.25) | 0.696 |  | 0.93 (0.71 - 1.22) | 0.593 |
|  |  |  |  |  |  |  |  |  |  |  |  |  |  |  |  |  |  |  |
| **Eggs** |  |  |  |  |  |  |  |  |  |  |  |  |  |  |  |  |  |  |
| low (0-1) |  | 148 |  | 422 |  | 35.1 |  | reference |  |  | reference |  |  | reference |  |  | reference |  |
| mod (2-4) |  | 133 |  | 377 |  | 35.3 |  | 1.01 (0.83 - 1.21) | 0.951 |  | 0.88 (0.72 - 1.08) | 0.223 |  | 0.87 (0.72 - 1.04) | 0.131 |  | 0.85 (0.71 - 1.03) | 0.104 |
| high (5-7) |  | 33 |  | 90 |  | 36.7 |  | 1.05 (0.77 - 1.41) | 0.772 |  | 0.89 (0.65 - 1.21) | 0.454 |  | 0.87 (0.65 - 1.16) | 0.334 |  | 0.88 (0.66 - 1.18) | 0.401 |
|  |  |  |  |  |  |  |  |  |  |  |  |  |  |  |  |  |  |  |
| **Soya products** |  |  |  |  |  |  |  |  |  |  |  |  |  |  |  |  |  |  |
| low (0-1) |  | 273 |  | 766 |  | 35.6 |  | reference |  |  | reference |  |  | reference |  |  | reference |  |
| mod (2-4) |  | 29 |  | 90 |  | 32.2 |  | 0.90 (0.66 - 1.24) | 0.530 |  | 0.85 (0.60 - 1.21) | 0.372 |  | 0.90 (0.66 - 1.22) | 0.501 |  | 0.87 (0.63 -1.22) | 0.425 |
| high (5-7) |  | 12 |  | 33 |  | 36.4 |  | 1.02 (0.64 - 1.62) | 0.932 |  | 0.97(0.58 - 1.60) | 0.899 |  | 0.84 (0.50 - 1.42) | 0.518 |  | 0.88 (0.53 -1.48) | 0.640 |
|  |  |  |  |  |  |  |  |  |  |  |  |  |  |  |  |  |  |  |
| **Chocolate** |  |  |  |  |  |  |  |  |  |  |  |  |  |  |  |  |  |  |
| low (0-1) |  | 103 |  | 296 |  | 34.8 |  | reference |  |  | reference |  |  | reference |  |  | reference |  |
| mod (2-4) |  | 149 |  | 404 |  | 36.9 |  | 1.06 (0.87 - 1.30) | 0.572 |  | 1.07 (0.86 - 1.32) | 0.545 |  | 1.16 (0.95 - 1.41) | 0.156 |  | 1.10 (0.92 -1.31) | 0.311 |
| high (5-7) |  | 62 |  | 189 |  | 32.8 |  | 0.94 (0.73 - 1.22) | 0.653 |  | 1.03 (0.77 - 1.37) | 0.850 |  | 1.10 (0.84 - 1.43) | 0.485 |  | 1.03 (0.82 -1.31) | 0.777 |
|  |  |  |  |  |  |  |  |  |  |  |  |  |  |  |  |  |  |  |
| **Nuts (almonds or walnuts)** |  |  |  |  |  |  |  |  |  |  |  |  |  |  |  |  |  |  |
| low (0-1) |  | 194 |  | 527 |  | 36.8 |  | reference |  |  | reference |  |  | reference |  |  | reference |  |
| mod (2-4) |  | 83 |  | 234 |  | 35.5 |  | 0.96 (0.78 - 1.18) | 0.724 |  | 0.84 (0.68 - 1.04) | 0.106 |  | 0.84 (0.69 - 1.03) | 0.087 |  | 0.83 (0.67 - 1.01) | 0.067 |
| high (5-7) |  | 37 |  | 128 |  | 28.9 |  | 0.79 (0.59 - 1.05) | 0.107 |  | 0.73 (0.54 - 0.98) | 0.039 |  | 0.78 (0.59 - 1.03) | 0.075 |  | 0.80 (0.61 - 1.06) | 0.115 |
|  |  |  |  |  |  |  |  |  |  |  |  |  |  |  |  |  |  |  |

**Footnotes**

^1^Analysis based on complete maternal and paternal diet dataset with no covariate adjustment

^2^Analysis based on complete maternal and paternal diet dataset with covariate adjustment (age at conception, BMI, ethnicity, smoking status at baseline, alcohol status at baseline, number of previous live births at baseline, number of previous miscarriages at baseline, linked paternal diet for each specific food group)

^3^Analysis based on multiple imputation dataset for missing maternal confounders and paternal dietary information with covariate adjustment

^4^Analysis based on multiple imputation dataset for missing maternal dietary information, maternal confounders, and paternal dietary information with covariate adjustment
