# Supplementary material for: The Association Between Periconceptual Maternal Dietary Patterns and Miscarriage Risk in Women With Recurrent Miscarriages: A Multicentre Cohort Study
Source: BJOG. 2024 Nov 26;132(4):504–17. doi: 10.1111/1471-0528.18022 (PMC11794061; doi:10.1111/1471-0528.18022)
Supplement: Supplementary file 5 — Table S2. [file BJO-132-504-s006.docx]

**Table S2.** Characteristics of Tommy’s Net Cohort with known pregnancy outcomes, stratified by maternal dietary data availability and inclusion in analysis

|  |  |  |  |  |  |  |  |  |  |  |  |  |  |  |
| --- | --- | --- | --- | --- | --- | --- | --- | --- | --- | --- | --- | --- | --- | --- |
| **Cohort characteristics** |  | **Cohort with complete maternal dietary data**^1^  *mean ± SD*  *number / total ( % )* | | | | | |  | **Cohort with missing maternal dietary data**^2^  *mean ± SD*  *number / total ( % )* | | | | | |
|  |  |  |  |  |  |  |  |  |  |  |  |  |  |  |
|  |  |  |  |  |  |  |  |  |  |  |  |  |  |  |
|  |  |  |  |  |  |  |  |  |  |  |  |  |  |  |
| Total number |  | 1035 | | | | | |  | 344 | | | | | |
|  |  |  |  |  |  |  |  |  |  |  |  |  |  |  |
| Maternal age at conception *(years)* |  |  |  | 34.3 | ± | 4.9 |  |  |  |  | 33.3 | ± | 5.8 |  |
| BMI *(kg/m^2^)* |  |  |  | 26.3 | ± | 5.5 |  |  |  |  | 27.1 | ± | 5.6 |  |
| White ethnicity |  | 890 | / | 1035 | ( | 86.0 | ) |  | 220 | / | 261 | ( | 84.3 | ) |
| Number of previous live birth at baseline |  |  |  | 0.5 | ± | 0.7 |  |  |  |  | 0.7 | ± | 0.9 |  |
| Number of previous miscarriages at baseline |  |  |  | 3.5 | ± | 1.7 |  |  |  |  | 3.2 | ± | 1.4 |  |
| Miscarriage at follow-up |  | 359 | / | 1035 | ( | 34.7 | ) |  | 164 | / | 334 | ( | 49.1 | ) |
|  |  |  |  |  |  |  |  |  |  |  |  |  |  |  |

**Footnotes**

^1^Cohort included in the primary analysis

^2^Cohort excluded from the primary analysis
