# Supplementary material for: The Association Between Periconceptual Maternal Dietary Patterns and Miscarriage Risk in Women With Recurrent Miscarriages: A Multicentre Cohort Study
Source: BJOG. 2024 Nov 26;132(4):504–17. doi: 10.1111/1471-0528.18022 (PMC11794061; doi:10.1111/1471-0528.18022)
Supplement: Supplementary file 6 — Table S3. [file BJO-132-504-s004.docx]

**Table S3.** Proportion of missing data in Tommy’s Net cohort with recurrent miscarriages by maternal food category exposure tertiles

| **Proportion of missing data** |  | | **Low intake**  **(0-1 days per week)** | | | | | | |  | | **Moderate intake**  **(2-4 days per week)** | | | | | | |  | | **High intake**  **(5-7 days per week)** | | | | | | |
| --- | --- | --- | --- | --- | --- | --- | --- | --- | --- | --- | --- | --- | --- | --- | --- | --- | --- | --- | --- | --- | --- | --- | --- | --- | --- | --- | --- |
|  |  | *number ( % )* | | | | | | |  | | *number ( % )* | | | | | | |  | | *number ( % )* | | | | | | |  |
| **Fresh fruit** total |  | | 77 | | |  |  |  | |  | | 296 | | |  |  |  | |  | | 662 | | |  |  |  | |
| Age at conception |  | | 0 | | | ( | 0.0 | ) | |  | | 0 | | | ( | 0.0 | ) | |  | | 0 | | | ( | 0.0 | ) | |
| BMI (kg/m^2^) |  | | 0 | | | ( | 0.0 | ) | |  | | 7 | | | ( | 2.4 | ) | |  | | 17 | | | ( | 2.6 | ) | |
| Ethnicity |  | | 0 | | | ( | 0.0 | ) | |  | | 0 | | | ( | 0.0 | ) | |  | | 0 | | | ( | 0.0 | ) | |
| Smoking status at baseline |  | | 0 | | | ( | 0.0 | ) | |  | | 1 | | | ( | 0.3 | ) | |  | | 1 | | | ( | 0.2 | ) | |
| Alcohol consumption status at baseline |  | | 0 | | | ( | 0.0 | ) | |  | | 1 | | | ( | 0.3 | ) | |  | | 0 | | | ( | 0.0 | ) | |
| Baseline obstetric history |  | | 0 | | | ( | 0.0 | ) | |  | | 0 | | | ( | 0.0 | ) | |  | | 0 | | | ( | 0.0 | ) | |
| Paternal fresh fruit consumption |  | | 17 | | | ( | 22.1 | ) | |  | | 40 | | | ( | 13.5 | ) | |  | | 89 | | | ( | 13.4 | ) | |
|  |  | |  | | |  |  |  | |  | |  | | |  |  |  | |  | |  | | |  |  |  | |
| **Fresh vegetables** total |  | | 42 | | |  |  |  | |  | | 226 | | |  |  |  | |  | | 767 | | |  |  |  | |
| Age at conception |  | | 0 | | | ( | 0.0 | ) | |  | | 0 | | | ( | 0.0 | ) | |  | | 0 | | | ( | 0.0 | ) | |
| BMI (kg/m^2^) |  | | 1 | | | ( | 2.4 | ) | |  | | 4 | | | ( | 1.8 | ) | |  | | 19 | | | ( | 2.5 | ) | |
| Ethnicity |  | | 0 | | | ( | 0.0 | ) | |  | | 0 | | | ( | 0.0 | ) | |  | | 0 | | | ( | 0.0 | ) | |
| Smoking status at baseline |  | | 0 | | | ( | 0.0 | ) | |  | | 0 | | | ( | 0.0 | ) | |  | | 2 | | | ( | 0.3 | ) | |
| Alcohol consumption status at baseline |  | | 0 | | | ( | 0.0 | ) | |  | | 0 | | | ( | 0.0 | ) | |  | | 1 | | | ( | 0.1 | ) | |
| Baseline obstetric history |  | | 0 | | | ( | 0.0 | ) | |  | | 0 | | | ( | 0.0 | ) | |  | | 0 | | | ( | 0.0 | ) | |
| Paternal fresh vegetables consumption |  | | 10 | | | ( | 23.8 | ) | |  | | 34 | | | ( | 15.0 | ) | |  | | 102 | | | ( | 13.3 | ) | |
|  |  | |  | | |  |  |  | |  | |  | | |  |  |  | |  | |  | | |  |  |  | |
| **Red meat** total |  | | 469 | | |  |  |  | |  | | 554 | | |  |  |  | |  | | 12 | | |  |  |  | |
| Age at conception |  | | 0 | | | ( | 0.0 | ) | |  | | 0 | | | ( | 0.0 | ) | |  | | 0 | | | ( | 0.0 | ) | |
| BMI (kg/m^2^) |  | | 9 | | | ( | 1.9 | ) | |  | | 15 | | | ( | 2.7 | ) | |  | | 0 | | | ( | 0.0 | ) | |
| Ethnicity |  | | 0 | | | ( | 0.0 | ) | |  | | 0 | | | ( | 0.0 | ) | |  | | 0 | | | ( | 0.0 | ) | |
| Smoking status at baseline |  | | 2 | | | ( | 0.4 | ) | |  | | 0 | | | ( | 0.0 | ) | |  | | 0 | | | ( | 0.0 | ) | |
| Alcohol consumption status at baseline |  | | 1 | | | ( | 0.2 | ) | |  | | 0 | | | ( | 0.0 | ) | |  | | 0 | | | ( | 0.0 | ) | |
| Baseline obstetric history |  | | 0 | | | ( | 0.0 | ) | |  | | 0 | | | ( | 0.0 | ) | |  | | 0 | | | ( | 0.0 | ) | |
| Paternal red meat consumption |  | | 71 | | | ( | 15.1 | ) | |  | | 73 | | | ( | 13.2 | ) | |  | | 2 | | | ( | 16.7 | ) | |
|  |  | |  |  |  |  |  |  | |  | |  |  |  |  |  |  | |  | |  | | |  |  |  | |
| **White meat** total |  | | 272 | | |  |  |  | |  | | 659 | | |  |  |  | |  | | 104 | | |  |  |  | |
| Age at conception |  | | 0 | | | ( | 0.0 | ) | |  | | 0 | | | ( | 0.0 | ) | |  | | 0 | | | ( | 0.0 | ) | |
| BMI (kg/m^2^) |  | | 7 | | | ( | 2.6 | ) | |  | | 14 | | | ( | 2.1 | ) | |  | | 3 | | | ( | 2.9 | ) | |
| Ethnicity |  | | 0 | | | ( | 0.0 | ) | |  | | 0 | | | ( | 0.0 | ) | |  | | 0 | | | ( | 0.0 | ) | |
| Smoking status at baseline |  | | 1 | | | ( | 0.4 | ) | |  | | 1 | | | ( | 0.2 | ) | |  | | 0 | | | ( | 0.0 | ) | |
| Alcohol consumption status at baseline |  | | 1 | | | ( | 0.4 | ) | |  | | 0 | | | ( | 0.0 | ) | |  | | 0 | | | ( | 0.0 | ) | |
| Baseline obstetric history |  | | 0 | | | ( | 0.0 | ) | |  | | 0 | | | ( | 0.0 | ) | |  | | 0 | | | ( | 0.0 | ) | |
| Paternal white meat consumption |  | | 47 | | | ( | 17.3 | ) | |  | | 88 | | | ( | 13.4 | ) | |  | | 11 | | | ( | 10.6 | ) | |
|  |  | |  |  |  |  |  |  | |  | |  |  |  |  |  |  | |  | |  |  |  |  |  |  | |
| **Fish** total |  | | 691 | | |  |  |  | |  | | 325 | | |  |  |  | |  | | 19 | | |  |  |  | |
| Age at conception |  | | 0 | | | ( | 0.0 | ) | |  | | 0 | | | ( | 0.0 | ) | |  | | 0 | | | ( | 0.0 | ) | |
| BMI (kg/m^2^) |  | | 16 | | | ( | 2.3 | ) | |  | | 7 | | | ( | 2.2 | ) | |  | | 1 | | | ( | 5.3 | ) | |
| Ethnicity |  | | 0 | | | ( | 0.0 | ) | |  | | 0 | | | ( | 0.0 | ) | |  | | 0 | | | ( | 0.0 | ) | |
| Smoking status at baseline |  | | 1 | | | ( | 0.1 | ) | |  | | 1 | | | ( | 0.3 | ) | |  | | 0 | | | ( | 0.0 | ) | |
| Alcohol consumption status at baseline |  | | 1 | | | ( | 0.1 | ) | |  | | 0 | | | ( | 0.0 | ) | |  | | 0 | | | ( | 0.0 | ) | |
| Baseline obstetric history |  | | 0 | | | ( | 0.0 | ) | |  | | 0 | | | ( | 0.0 | ) | |  | | 0 | | | ( | 0.0 | ) | |
| Paternal fish consumption |  | | 91 | | | ( | 13.2 | ) | |  | | 52 | | | ( | 16.0 | ) | |  | | 3 | | | ( | 15.8 | ) | |
|  |  | |  | | |  |  |  | |  | |  | | |  |  |  | |  | |  | | |  |  |  | |
| **Dairy products** total |  | | 210 | | |  |  |  | |  | | 168 | | |  |  |  | |  | | 657 | | |  |  |  | |
| Age at conception |  | | 0 | | | ( | 0.0 | ) | |  | | 0 | | | ( | 0.0 | ) | |  | | 0 | | | ( | 0.0 | ) | |
| BMI (kg/m^2^) |  | | 2 | | | ( | 1.0 | ) | |  | | 5 | | | ( | 3.0 | ) | |  | | 17 | | | ( | 2.6 | ) | |
| Ethnicity |  | | 0 | | | ( | 0.0 | ) | |  | | 0 | | | ( | 0.0 | ) | |  | | 0 | | | ( | 0.0 | ) | |
| Smoking status at baseline |  | | 0 | | | ( | 0.0 | ) | |  | | 0 | | | ( | 0.0 | ) | |  | | 2 | | | ( | 0.3 | ) | |
| Alcohol consumption status at baseline |  | | 0 | | | ( | 0.0 | ) | |  | | 0 | | | ( | 0.0 | ) | |  | | 1 | | | ( | 0.2 | ) | |
| Baseline obstetric history |  | | 0 | | | ( | 0.0 | ) | |  | | 0 | | | ( | 0.0 | ) | |  | | 0 | | | ( | 0.0 | ) | |
| Paternal dairy products consumption |  | | 34 | | | ( | 16.2 | ) | |  | | 28 | | | ( | 16.7 | ) | |  | | 84 | | | ( | 12.8 | ) | |
|  |  | |  |  |  |  |  |  | |  | |  |  |  |  |  |  | |  | |  |  |  |  |  |  | |
| **Eggs** total |  | | 497 | | |  |  |  | |  | | 433 | | |  |  |  | |  | | 105 | | |  |  |  | |
| Age at conception |  | | 0 | | | ( | 0.0 | ) | |  | | 0 | | | ( | 0.0 | ) | |  | | 0 | | | ( | 0.0 | ) | |
| BMI (kg/m^2^) |  | | 9 | | | ( | 1.8 | ) | |  | | 11 | | | ( | 2.5 | ) | |  | | 4 | | | ( | 3.8 | ) | |
| Ethnicity |  | | 0 | | | ( | 0.0 | ) | |  | | 0 | | | ( | 0.0 | ) | |  | | 0 | | | ( | 0.0 | ) | |
| Smoking status at baseline |  | | 1 | | | ( | 0.2 | ) | |  | | 1 | | | ( | 0.2 | ) | |  | | 0 | | | ( | 0.0 | ) | |
| Alcohol consumption status at baseline |  | | 0 | | | ( | 0.0 | ) | |  | | 1 | | | ( | 0.2 | ) | |  | | 0 | | | ( | 0.0 | ) | |
| Baseline obstetric history |  | | 0 | | | ( | 0.0 | ) | |  | | 0 | | | ( | 0.0 | ) | |  | | 0 | | | ( | 0.0 | ) | |
| Paternal eggs consumption |  | | 75 | | | ( | 15.1 | ) | |  | | 56 | | | ( | 12.9 | ) | |  | | 15 | | | ( | 14.3 | ) | |
|  |  | |  |  |  |  |  |  | |  | |  |  |  |  |  |  | |  | |  |  |  |  |  |  | |
| **Soya products** total |  | | 896 | | |  |  |  | |  | | 102 | | |  |  |  | |  | | 37 | | |  |  |  | |
| Age at conception |  | | 0 | | | ( | 0.0 | ) | |  | | 0 | | | ( | 0.0 | ) | |  | | 0 | | | ( | 0.0 | ) | |
| BMI (kg/m^2^) |  | | 19 | | | ( | 2.1 | ) | |  | | 4 | | | ( | 3.9 | ) | |  | | 1 | | | ( | 2.7 | ) | |
| Ethnicity |  | | 0 | | | ( | 0.0 | ) | |  | | 0 | | | ( | 0.0 | ) | |  | | 0 | | | ( | 0.0 | ) | |
| Smoking status at baseline |  | | 1 | | | ( | 0.1 | ) | |  | | 1 | | | ( | 1.0 | ) | |  | | 0 | | | ( | 0.0 | ) | |
| Alcohol consumption status at baseline |  | | 0 | | | ( | 0.0 | ) | |  | | 1 | | | ( | 1.0 | ) | |  | | 0 | | | ( | 0.0 | ) | |
| Baseline obstetric history |  | | 0 | | | ( | 0.0 | ) | |  | | 0 | | | ( | 0.0 | ) | |  | | 0 | | | ( | 0.0 | ) | |
| Paternal soya products consumption |  | | 130 | | | ( | 14.5 | ) | |  | | 12 | | | ( | 11.8 | ) | |  | | 4 | | | ( | 10.8 | ) | |
|  |  | |  |  |  |  |  |  | |  | |  |  |  |  |  |  | |  | |  |  |  |  |  |  | |
| **Chocolate** total |  | | 356 | | |  |  |  | |  | | 456 | | |  |  |  | |  | | 223 | | |  |  |  | |
| Age at conception |  | | 0 | | | ( | 0.0 | ) | |  | | 0 | | | ( | 0.0 | ) | |  | | 0 | | | ( | 0.0 | ) | |
| BMI (kg/m^2^) |  | | 6 | | | ( | 1.7 | ) | |  | | 11 | | | ( | 2.4 | ) | |  | | 7 | | | ( | 3.1 | ) | |
| Ethnicity |  | | 0 | | | ( | 0.0 | ) | |  | | 0 | | | ( | 0.0 | ) | |  | | 0 | | | ( | 0.0 | ) | |
| Smoking status at baseline |  | | 0 | | | ( | 0.0 | ) | |  | | 2 | | | ( | 0.4 | ) | |  | | 0 | | | ( | 0.0 | ) | |
| Alcohol consumption status at baseline |  | | 0 | | | ( | 0.0 | ) | |  | | 1 | | | ( | 0.2 | ) | |  | | 0 | | | ( | 0.0 | ) | |
| Baseline obstetric history |  | | 0 | | | ( | 0.0 | ) | |  | | 0 | | | ( | 0.0 | ) | |  | | 0 | | | ( | 0.0 | ) | |
| Paternal chocolate consumption |  | | 60 | | | ( | 16.9 | ) | |  | | 52 | | | ( | 11.4 | ) | |  | | 34 | | | ( | 15.2 | ) | |
|  |  | |  |  |  |  |  |  | |  | |  |  |  |  |  |  | |  | |  |  |  |  |  |  | |
| **Nuts (almonds or walnuts)** total |  | | 613 | | |  |  |  | |  | | 270 | | |  |  |  | |  | | 152 | | |  |  |  | |
| Age at conception |  | | 0 | | | ( | 0.0 | ) | |  | | 0 | | | ( | 0.0 | ) | |  | | 0 | | | ( | 0.0 | ) | |
| BMI (kg/m^2^) |  | | 14 | | | ( | 2.3 | ) | |  | | 7 | | | ( | 2.6 | ) | |  | | 3 | | | ( | 2.0 | ) | |
| Ethnicity |  | | 0 | | | ( | 0.0 | ) | |  | | 0 | | | ( | 0.0 | ) | |  | | 0 | | | ( | 0.0 | ) | |
| Smoking status at baseline |  | | 0 | | | ( | 0.0 | ) | |  | | 2 | | | ( | 0.7 | ) | |  | | 0 | | | ( | 0.0 | ) | |
| Alcohol consumption status at baseline |  | | 0 | | | ( | 0.0 | ) | |  | | 1 | | | ( | 0.4 | ) | |  | | 0 | | | ( | 0.0 | ) | |
| Baseline obstetric history |  | | 0 | | | ( | 0.0 | ) | |  | | 0 | | | ( | 0.0 | ) | |  | | 0 | | | ( | 0.0 | ) | |
| Paternal nuts consumption |  | | 86 | | | ( | 14.0 | ) | |  | | 36 | | | ( | 13.3 | ) | |  | | 24 | | | ( | 15.8 | ) | |
