# Supplementary material for: The Association Between Periconceptual Maternal Dietary Patterns and Miscarriage Risk in Women With Recurrent Miscarriages: A Multicentre Cohort Study
Source: BJOG. 2024 Nov 26;132(4):504–17. doi: 10.1111/1471-0528.18022 (PMC11794061; doi:10.1111/1471-0528.18022)
Supplement: Supplementary file 7 — Table S4. [file BJO-132-504-s007.docx]

**Table S4.** Poisson regression analysis of overall diet in Tommy’s Net recurrent miscarriage cohort by tertiles of data-derived dietary pattern scores, with Bootstrapping

|  |  |  |  |  |  |  |  |  |  |  |  |  |  |  |  |  |
| --- | --- | --- | --- | --- | --- | --- | --- | --- | --- | --- | --- | --- | --- | --- | --- | --- |
| Dietary pattern with focus on specified components |  | RR (95% CI)  Univariable^1^ | | | | | |  |  | RR (95% CI)  Multivariable^2^ | | | | | |  |
|  |  |  |  |  |  |  |  | *p*-value |  |  |  |  |  |  |  | *p*-value |
|  |  |  |  |  |  |  |  |  |  |  |  |  |  |  |  |  |
| Fresh Fruit and Vegetables |  |  |  |  |  |  |  |  |  |  |  |  |  |  |  |  |
| T1 |  | reference | | | | | | |  | reference | | | | | | |
| T2 |  | 0.83 | ( | 0.66 | - | 1.40 | ) | 0.10 |  | 0.86 | ( | 0.68 | - | 2.54 | ) | 0.23 |
| T3 |  | 0.89 | ( | 0.81 | - | 1.21 | ) | 0.91 |  | 0.58 | ( | 0.76 | - | 1.18 | ) | 0.62 |
|  |  |  |  |  |  |  |  |  |  |  |  |  |  |  |  |  |
| Fish and Eggs |  |  |  |  |  |  |  |  |  |  |  |  |  |  |  |  |
| T1 |  | reference | | | | | | |  | reference | | | | | | |
| T2 |  | 0.98 | ( | 0.79 | - | 1.22 | ) | 0.84 |  | 0.94 | ( | 0.74 | - | 1.20 | ) | 0.62 |
| T3 |  | 0.95 | ( | 0.75 | - | 1.21 | ) | 0.69 |  | 0.88 | ( | 0.88 | - | 1.23 | ) | 0.47 |
|  |  |  |  |  |  |  |  |  |  |  |  |  |  |  |  |  |

**Footnotes**

T, tertile; RR, risk ratio; CI, confidence interval.

T1: Lowest scoring tertile based on a data-derived dietary pattern focused on specified components, T2: Middle tertile, T3: Highest scoring tertile.

^1^No covariate adjustment

^2^Covariate adjustment for maternal factors: age at conception, BMI, ethnicity, smoking status at baseline, alcohol status at baseline, number of previous live births at baseline, number of previous miscarriages at baseline
